# MMP11 and CD2 as novel prognostic factors in hormone receptor-negative, HER2-positive breast cancer

**DOI:** 10.1007/s10549-017-4234-4

**Published:** 2017-04-13

**Authors:** Jinil Han, Yoon-La Choi, Haein Kim, Jun Young Choi, Se Kyung Lee, Jeong Eon Lee, Joon-Seok Choi, Sarah Park, Jong-Sun Choi, Young Deug Kim, Seok Jin Nam, Byung-Ho Nam, Mi Jeong Kwon, Young Kee Shin

**Affiliations:** 1 Gencurix, Inc., Seoul, 08394 Korea; 2 Laboratory of Cancer Genomics and Molecular Pathology, Samsung Medical Center, Sungkyunkwan University School of Medicine, Seoul, 06356 Korea; 3 Department of Pathology and Translational Genomics, Samsung Medical Center, Sungkyunkwan University School of Medicine, Seoul, 06356 Korea; 4 Department of Health Sciences and Technology, SAIHST, Sungkyunkwan University, Seoul, 06356 Korea; 5 Laboratory of Molecular Pathology and Cancer Genomics, Department of Pharmacy, College of Pharmacy, Seoul National University, 1 Gwanak-ro, Gwanak-gu, Seoul, 08826 Korea; 6 Department of Surgery, Samsung Medical Center, Sungkyunkwan University School of Medicine, Seoul, 06356 Korea; 7 College of Pharmacy, Catholic University of Daegu, Gyeongbuk, 38430 Korea; 8 The Center for Anti-cancer Companion Diagnostics, Bio-MAX/N-Bio, Seoul National University, Seoul, 08826 Korea; 9 R&D center, ABION Inc., Seoul, 08394 Korea; 10 Department of Cancer Control and Policy, Graduate School of Cancer Science and Policy, National Cancer Center, Goyang, Gyeonggi-do 10408 Korea; 11 HERINGS, The Institute of Advanced Clinical and Biomedical Research, Seoul, 06051 Korea; 12 College of Pharmacy, Kyungpook National University, 80 Daehak-ro, Buk-gu, Daegu, 41566 Korea; 13 Research Institute of Pharmaceutical Sciences, College of Pharmacy, Kyungpook National University, Daegu, 41566 Korea; 14 Department of Molecular Medicine and Biopharmaceutical Sciences, Graduate School of Convergence Science and Technology, Seoul National University, Seoul, 08826 Korea

**Keywords:** Breast cancer, Hormone receptor-negative, HER2-positive (HR**−**/HER2+) breast cancer, Prognostic model, Risk of distant metastasis, Immune response-related genes (i-genes), Proliferation-related genes (p-genes)

## Abstract

**Purpose:**

More accurate prediction of patient outcome based on molecular subtype is required to identify patients who will benefit from specific treatments.

**Methods:**

We selected novel 16 candidate prognostic genes, including 10 proliferation-related genes (p-genes) and 6 immune response-related genes (i-genes), from the gene list identified in our previous study. We then analyzed the association between their expression, measured by quantitative real-time reverse transcription-PCR in formalin-fixed, paraffin-embedded tissues, and clinical outcome in 819 breast cancer patients according to molecular subtype.

**Results:**

The prognostic significance of clinical and gene variables varied according to the molecular subtype. Univariate analysis showed that positive lymph node status was significantly correlated with the increased risk of distant metastasis in all subtypes except the hormone receptor-negative, HER2-positive (HR−/HER2**+**) subtype. Most p-genes were significantly associated with poor prognosis in patients with the HR**+**/HER2− subtype, whereas i-genes correlated with a favorable outcome in patients with HR−/HER2**+** breast cancer. In HR−/HER2+ breast cancer, four genes (three i-genes *BTN3A2*, *CD2*, and *TRBC1* and the p-gene *MMP11*) were significantly associated with distant metastasis-free survival (DMFS). A new prognostic model for HR−/HER2+ breast cancer based on the expression of *MMP11* and *CD2* was developed and the DMFS for patients in the high-risk group according to our model was significantly lower than that for those in the low-risk group. Multivariate analyses revealed that our risk score is an independent prognostic factor for DMFS. Moreover, C-index showed that our risk score has a superior prognostic performance to traditional clinicopathological factors.

**Conclusions:**

Our new prognostic model for HR−/HER2+ breast cancer provides more accurate information on the risk of distant metastasis than traditional clinical prognostic factors and may be used to identify patients with a good prognosis in this aggressive subtype of breast cancer.

**Electronic supplementary material:**

The online version of this article (doi:10.1007/s10549-017-4234-4) contains supplementary material, which is available to authorized users.

## Introduction

Breast cancer is a highly heterogeneous disease and is currently classified into four general molecular subtypes according to the status of hormone receptors, including estrogen receptor (ER) or progesterone receptor (PR), and human epidermal growth factor receptor 2 (HER2) [1]. Each subtype has distinct molecular characteristics and although individual patient prognosis varies, patients with the hormone receptor-positive, HER2-negative (HR+/HER2**−**) subtype generally have a more favorable prognosis whereas those with hormone receptor-negative (HR**−)** breast cancer have a poor prognosis [2–4]. Because treatment strategies for breast cancer are dependent on molecular subtype and patient prognosis, it is important to identify specific prognostic biomarkers for each molecular subtype to determine appropriate treatments.

Gene expression-based approaches provide significant prognostic or predictive information, and commercial assays such as Oncotype DX [5, 6], MammaPrint [7, 8], Prosigna [9, 10], and EndoPredict [11] based on multigene expression profiling in frozen or formalin-fixed, paraffin-embedded (FFPE) samples have been developed for ER-positive (ER+) breast cancer. These assays predict the risk of distant recurrence after hormone therapy and are useful to identify patients who will benefit from adjuvant chemotherapy by discriminating high- and low-risk patients with early ER+ breast cancer. However, there are certain limitations to the currently available assays that use multigene expression signatures based on proliferation-related genes (p-genes) including diminished prognostic ability to predict the late distant recurrence (beyond 5 years from diagnosis or primary treatment). Furthermore, commercialized kits based on various multigene predictors of clinical outcome are prognostic only for HR+ subtypes, whereas there is no available commercial assay for HR**−** breast cancer. Meta-analysis using publicly available microarray data from over 2100 patients showed that the key biological processes associated with the clinical outcome of patients with breast cancer differs according to the molecular subtype [12]. This study selected seven prototype genes (*AURKA*, *PLAU*, *STAT1*, *VEGF*, *CASP3*, *ESR1*, and *ERBB2*) representing different biological processes, proliferation, tumor invasion/metastasis, immune response, angiogenesis, apoptosis phenotypes, and ER and HER2 signaling, respectively, and assessed the association between the expression of these seven gene modules and clinical variables and relapse-free survival of patients in each subtype of the breast cancer. The results showed that the prognostic performance of the proliferation module was limited to the ER+/HER2**−** subgroup, and genes associated with tumor invasion and immune response have prognostic value in ER**−**/HER2**−** or ER**−**/HER2+ subtypes. Recent studies reporting prognostic genes or gene signatures predicting recurrence or distant metastasis for HR**−** breast cancer [13–16] further confirm that expression of immune response-related genes (i-genes) is primarily associated with good clinical outcome in patients with HR**−** breast cancer, unlike the strong prognostic significance of p-genes, which predict recurrence in HR+ breast cancer. However, these results are based mainly on gene expression microarray data, and validation of most of the identified prognostic genes or signatures has not been performed.

We previously identified 384 candidate prognostic genes associated with distant metastasis in patients with lymph node-negative (LN**−**) early breast cancer using public microarray gene expression data [17]. This study aimed to identify novel prognostic genes associated with the risk of distant metastasis in patients with various subtypes of breast cancer from the candidate list established in our previous study. We validated the expression of 16 candidate prognostic genes by performing quantitative real-time reverse transcription-PCR (qRT-PCR) in a large number of FFPE tissue samples, and we then assessed the association between their expression and the risk of distant metastasis in 819 patients with breast cancer. Based on the resulting set of significant prognostic genes, we developed a prognostic model to predict the risk of distant metastasis in HR−/HER2+ breast cancer.

## Materials and methods

### Ethical statement

This study was approved by the Institutional Review Board (IRB) of the Samsung Medical Center (SMC) (Seoul, Korea) and performed in accordance with the Declaration of Helsinki. The study was retrospective and informed consents from the patients involved in the study were not required, as per the guidelines of the IRB. Patient information was anonymized and de-identified prior to analysis.

### Study population

Our study adhered to the Reporting Recommendations for Tumor Marker Prognostic Studies (REMARK) criteria in the design, analysis, and interpretation of the results [18]. A total of 997 FFPE tissue specimens were obtained from patients with breast cancer who underwent curative resection for primary tumors with LN dissection at the SMC between 1994 and 2002. We also obtained 50 frozen tissue samples paired with FFPE samples from the same patients. Detailed inclusion/exclusion criteria for tissue samples are described in Supplementary materials and methods. Molecular subtypes of breast cancer were categorized as HR+/HER2**−** (ER+ or PR+/HER2**−**), HR+/HER2+ (ER+ or PR+/HER2+), HR−/HER2+ (ER**−**/PR**−**/HER2+), or triple-negative breast cancer (TNBC, ER**−**/PR**−**/HER2**−**) according to the expression status of ER, PR, and HER2, as classified in our previous study [19].

### Selection of candidate prognostic genes

From 384 candidate genes identified in our previous study using public gene expression microarray data [17], a total of 30 candidate genes were selected based on the following criteria: (1) high correlation with either proliferation or immune response, (2) high variability between samples (large interquartile range), and (3) high mean expression value. Based on the results of qRT-PCR, 16 genes with high correlation of expression between FFPE and frozen tissues were further selected. For details, see Supplementary materials and methods.

### qRT-PCR and normalization of qRT-PCR data

RNA extraction and qRT-PCR were performed as described in Supplementary materials and methods. The relative expression value of each gene was calculated based on the difference between the average Cq value of the three reference genes (*CTBP1*, *CUL1*, and *UBQLN1*) and the target Cq value for each sample:

1 $$ \Delta C_{\text{q}} \_{\text{target}} = \left( {\left( {C_{\text{q}} \_CTBP1 + C_{\text{q}} \_CUL1 + C_{\text{q}} \_UBQLN1} \right)/ 3} \right) - C_{\text{q}} \_{\text{target}} + 30 $$

### Development of the prognostic model for HR−/HER2+ breast cancer and cross validation

Based on stepwise multivariate analyses results, a prognostic model to predict the risk of distant metastasis in HR**−**/HER2+ breast cancer was developed. Relative expression values of the two prognostic genes normalized by the average expression level of three reference genes were used to calculate the risk score, a molecular predictor of distant metastasis. The risk score was defined as follows:

2 $$ {\text{Risk score}} = 0. 4 5\times \Delta C_{\text{q}} \_MMP11 - 0. 4 8\times \Delta C_{\text{q}} \_CD2 $$

Higher values indicate a higher risk of distant metastasis. For development and performance evaluation of the prognostic model, a 10-fold cross validation procedure was used [20].

We compared the prognostic performance of our prognostic model with other prognostic models based on clinical variables, including The Nottingham prognostic index (NPI) score [21] and two web-based prediction tools, SNAP (www.CancerMath.net) [22] and PREDICT (www.predict.nhs.uk) [23, 24]. The Harrell’s concordance index (C-index) [25] was calculated to estimate the discrimination capability of each prognostic model and to compare their prognostic performance. Detailed information on the development of the prognostic model is provided in the Supplementary materials and methods.

### Statistical analyses

Distant metastasis-free survival (DMFS) was defined as the time from the date of surgery for the primary tumor to the date of distant metastasis. Overall survival (OS) and Disease-free survival (DFS) were defined as described in our previous study [26]. Univariate and multivariate analyses were performed using Cox proportional hazard model. For univariate and multivariate analyses, missing* C*_q_ values were imputed using the algorithm developed by McCall et al. [27]. In particular, selected variables in univariate analysis were entered in a stepwise multivariate Cox proportional hazard model to determine independent contributions of predictors for the primary endpoint. Probability of distant metastasis was estimated by the Kaplan–Meier method and the log-rank test was used to test the differences in survival between the groups. Differences were to be considered statistically significant if the *P* value was less than 0.05. All statistical analyses were performed using R 3.2.0 (http://r-project.org).

## Results

### Patient characteristics

Of the 997 FFPE tissue samples, histologically ineligible samples or those with an insufficient amount of tissue were excluded, as were samples that produced an insufficient amount of RNA. Gene expression was measured in a total of 926 FFPE samples by qRT-PCR. Cases with missing *C*q values for reference genes in the qRT-PCR data or with missing clinical information were further excluded, resulting in a total of 819 breast cancer patients with informative clinical data that were finally included in the analysis. The median patient age was 47.3 years (range 23.8–81.2), and the mean tumor size was 2.8 ± 1.6 cm (mean ± SD). Of the 819 patients, 51.6% (423/819) were LN**−**, whereas 48.4% (396/819) were LN+. A total of 86.3% (707/819) of the patients received adjuvant chemotherapy. The details on the clinicopathological characteristics of breast cancer patients grouped by molecular subtypes are illustrated in Table 1. Of the 819 cases, the majority comprised HR+ tumors, including HR+/HER2**−** (50.1%, 410/819) and HR+/HER2+ (13.7%, 112/819) subtypes. The HR+/HER2**−** subtype had the highest percent of histologic grade 1 and 2 tumors, whereas the HR**−**/HER2+ and TNBC subtypes consisted of a higher proportion of grade 3 tumors.

**Table 1:** Clinical characteristics of the breast cancer patients in this study

	Total (*n* = 819)	HR+/HER2− (*n* = 410)	HR+/HER2+ (*n* = 112)	HR−/HER2+ (*n* = 105)	TNBC (*n* = 192)
	No. (%)	No. (%)	No. (%)	No. (%)	No. (%)
Median age (min–max) (years)	47.3 (23.8–81.2)	47.3 (25.2–80.5)	45.5 (26.0–77.4)	52.8 (24.3–77.8)	46.0 (23.8–81.2)
Age (years)					
<50	486 (59.3)	241 (58.8)	76 (67.9)	43 (41.0)	126 (65.6)
≥50	333 (40.7)	169 (41.2)	36 (32.1)	62 (59.0)	66 (34.4)
Tumor size (cm)					
≤2	325 (39.7)	183 (44.6)	39 (34.8)	32 (30.5)	71 (37.0)
2–5	438 (53.5)	204 (49.8)	65 (58.0)	63 (60.0)	106 (55.2)
>5	56 (6.8)	23 (5.6)	8 (7.1)	10 (9.5)	15 (7.8)
Lymph node status					
Negative	423 (51.6)	203 (49.5)	45 (40.2)	59 (56.2)	116 (60.4)
Positive	396 (48.4)	207 (50.5)	67 (59.8)	46 (43.8)	76 (39.6)
pN					
0	423 (51.7)	203 (49.5)	45 (40.2)	59 (56.2)	116 (60.4)
1	214 (26.1)	112 (27.3)	40 (35.7)	22 (21.0)	40 (20.8)
2	97 (11.8)	51 (12.4)	16 (14.3)	11 (10.5)	19 (9.9)
3	85 (10.4)	44 (10.7)	11 (9.8)	13 (12.4)	17 (8.9)
Pathologic stage					
I	204 (24.9)	113 (27.6)	17 (15.2)	20 (19.0)	54 (28.1)
II	417 (50.9)	197 (48.0)	64 (57.1)	58 (55.2)	98 (51.0)
III	198 (24.2)	100 (24.4)	31 (27.7)	27 (25.7)	40 (20.8)
Histologic grade					
1	93 (11.4)	77 (18.8)	8 (7.1)	4 (3.8)	4 (2.1)
2	300 (36.6)	199 (48.5)	37 (33.0)	24 (22.9)	40 (20.8)
3	366 (44.7)	123 (30)	59 (52.7)	66 (62.9)	118 (61.5)
Unknown	60 (7.3)	11 (2.7)	8 (7.1)	11 (10.5)	30 (15.6)
Nuclear grade					
1	81 (9.9)	60 (14.6)	7 (6.3)	2 (1.9)	12 (6.3)
2	402 (49.1)	256 (62.4)	55 (49.1)	35 (33.3)	56 (29.2)
3	307 (37.5)	81 (19.8)	46 (41.1)	65 (61.9)	115 (59.9)
Unknown	29 (3.5)	13 (3.2)	4 (3.6)	3 (2.9)	9 (4.7)
Hormone therapy					
No	301 (36.8)	21 (5.1)	11 (9.8)	96 (91.4)	173 (90.1)
Yes	508 (62.0)	379 (92.4)	101 (90.2)	9 (8.6)	19 (9.9)
Unknown	10 (1.2)	10 (2.4)	0 (0.0)	0 (0.0)	0 (0.0)
Chemotherapy					
No	110 (13.4)	68 (16.6)	14 (12.5)	14 (13.3)	14 (7.3)
Yes	707 (86.3)	342 (83.4)	97 (86.6)	90 (85.7)	178 (92.7)
Unknown	2 (0.3)	0 (0.0)	1 (0.9)	1 (1.0)	0 (0.0)
Radiotherapy					
No	352 (43.0)	162 (39.5)	50 (44.6)	59 (56.2)	81 (42.2)
Yes	465 (56.8)	247 (60.2)	61 (54.5)	46 (43.8)	111 (57.8)
Unknown	2 (0.2)	1 (0.2)	1 (0.9)	0 (0.0)	0 (0.0)
NPI					
1	149 (18.2)	113 (27.6)	14 (12.5)	7 (6.7)	15 (7.8)
2	210 (25.6)	113 (27.6)	23 (20.5)	28 (26.7)	46 (24.0)
3	286 (34.9)	113 (27.6)	51 (45.5)	44 (41.9)	78 (40.6)
4	113 (13.8)	58 (14.1)	17 (15.2)	15 (14.3)	23 (12.0)
Unknown	61 (7.5)	13 (3.2)	7 (6.3)	11 (10.5)	30 (15.6)

*HR* hormone receptor, *HER2* human epidermal growth factor receptor 2, *TNBC* triple-negative breast cancer, *pN* pathologic nodal status, *NPI* Nottingham prognostic index

Kaplan–Meier curves for DMFS, DFS, and OS were generated according to molecular subtype. The median follow-up durations for DMFS, DFS, and OS were 9.68 (range 0.04–19.46), 9.45 (range 0.04–19.46), and 10.33 years (range 0.05–19.46), respectively. During this entire follow-up period, there were no significant differences in patient survival between molecular subtypes (Supplementary Fig. S1A). However, in terms of 5-year OS and DFS, significant (*P* < 0.001 for OS) or marginally significant (*P* = 0.069 for DFS) differences in patient survival between molecular subtypes were observed (Supplementary Fig. S1B). Patients with HR−/HER2+ subtype showed poorer 5-year survival than those with the HR+/HER2− subtype (Supplementary Fig. S1B).

### Univariate analysis of clinical variables according to molecular subtype

First, we analyzed the association of traditional clinicopathological factors with clinical outcome according to molecular subtype. Univariate analysis for DMFS showed that clinical variables such as larger tumor size, positive LN (LN+) involvement, and higher histologic grade were significantly associated with an increased risk of distant metastasis in the HR+/HER2**−** subtype (Table 2). However, only LN status was significantly correlated with DMFS in HR+/HER2+ cancers, whereas tumor size and LN status were significant for DMFS in TNBC. The significance of tumor size was limited to HER2**−** subtypes, including HR+/HER2**−** and TNBC. Of note, none of the clinical variables were significant in HR**−**/HER2+ breast cancer.

**Table 2:** Univariate analysis of clinical variables for DMFS according to molecular subtype

	Total				HR+/HER2−				HR+/HER2+				HR−/HER2+				TNBC			
	Hazard ratio	95% CI		*P* value	Hazard ratio	95% CI		*P* value	Hazard ratio	95% CI		*P* value	Hazard ratio	95% CI		*P* value	Hazard ratio	95% CI		*P* value
No. of patients (no. of events)		815 (209)				408 (108)				111 (32)				104 (28)				192 (41)		
Age (years)																				
<50	1.00				1.00				1.00				1.00				1.00			
≥50	0.74	0.56	0.99	**0.042**	0.76	0.51	1.14	0.184	0.47	0.19	1.15	0.098	0.83	0.39	1.76	0.630	0.76	0.39	1.50	0.431
Tumor size (cm)																				
≤2	1.00				1.00				1.00				1.00				1.00			
2–5	1.70	1.25	2.30	**0.001**	1.59	1.06	2.40	**0.025**	1.13	0.53	2.43	0.745	2.39	0.90	6.35	0.081	2.18	1.02	4.63	**0.044**
>5	2.13	1.26	3.60	**0.005**	2.25	1.08	4.66	**0.029**	1.05	0.23	4.82	0.947	1.68	0.32	8.64	0.538	3.91	1.31	11.68	**0.015**
Lymph node status																				
Negative	1.00				1.00				1.00				1.00				1.00			
Positive	2.74	2.04	3.68	**<0.001**	3.04	1.99	4.66	**<0.001**	2.78	1.20	6.42	**0.017**	1.87	0.88	3.96	0.101	2.82	1.51	5.29	**0.001**
Histologic grade																				
1	1.00				1.00				1.00				–				–			
2	1.64	0.90	2.97	0.104	1.56	0.83	2.94	0.169	1.13	0.13	9.64	0.914	1.00				1.00			
3	2.40	1.35	4.26	**0.003**	2.33	1.22	4.44	**0.010**	3.89	0.52	28.90	0.184	1.05	0.41	2.65	0.925	1.36	0.59	3.14	0.468

Hazard ratios with *P* values < 0.05 are marked in bold

*HR* hormone receptor, *HER2* human epidermal growth factor receptor 2, *TNBC* triple-negative breast cancer, *CI* confidence interval

Univariate analysis results for DFS and OS were similar to those for DMFS (Supplementary Table S1). None of the clinical variables were significantly associated with DFS or OS in HR**−**/HER2+ subtype tumors. In HR+/HER2+ tumors, younger age (age < 50) and LN+ status showed a significant association with increased risk of recurrence, whereas none of the clinical variables were significant for OS.

### Univariate analysis of gene variables according to molecular subtype

Univariate analysis for gene variables showed that the association between expression of each of the 16-candidate prognostic genes and distant metastasis differed according to molecular subtype. Most p-genes showed a significant association with DMFS in the HR+/HER2**−** subtype. High level expression of nine p-genes correlated significantly with a greater risk of distant metastasis in this subtype (Table 3).

**Table 3:** Univariate analysis of gene variables for DMFS according to molecular subtype

	All				HR+/HER2−				HR+/HER2+				HR−/HER2+				TNBC			
	Hazard ratio	95% CI		*P* value	Hazard ratio	95% CI		*P* value	Hazard ratio	95% CI		*P* value	Hazard ratio	95% CI		*P* value	Hazard ratio	95% CI		*P* value
No. of patients (no. of events)		815 (209)				408 (108)				111 (32)				104 (28)				192 (41)		
p-genes																				
*AURKA*	1.07	0.99	1.15	0.086	1.16	1.04	1.29	**0.006**	1.02	0.84	1.24	0.806	1.21	0.99	1.47	0.067	0.88	0.75	1.04	0.140
*CCNB2*	1.12	0.98	1.28	0.096	1.32	1.09	1.60	**0.005**	1.07	0.76	1.50	0.710	0.99	0.64	1.54	0.976	0.93	0.64	1.35	0.697
*FOXM1*	1.17	1.03	1.33	**0.015**	1.37	1.14	1.65	**0.001**	1.24	0.83	1.85	0.294	1.04	0.61	1.75	0.898	1.05	0.75	1.47	0.763
*MKI67*	1.19	1.03	1.38	**0.017**	1.36	1.11	1.66	**0.002**	1.22	0.86	1.73	0.270	1.12	0.76	1.64	0.578	0.95	0.65	1.38	0.772
*MMP11*	1.27	1.15	1.40	**<0.001**	1.22	1.06	1.39	**0.004**	1.39	1.07	1.80	**0.012**	1.57	1.16	2.13	**0.003**	1.16	0.92	1.46	0.208
*PTTG1*	1.02	0.85	1.21	0.853	1.02	0.79	1.32	0.896	1.25	0.84	1.86	0.267	0.97	0.57	1.66	0.917	0.93	0.60	1.43	0.736
*RACGAP1*	1.14	0.99	1.32	0.078	1.24	1.02	1.51	**0.028**	1.27	0.82	1.96	0.282	1.10	0.71	1.71	0.677	0.94	0.66	1.35	0.751
*RRM2*	1.17	1.02	1.35	**0.026**	1.40	1.14	1.71	**0.001**	1.65	1.07	2.53	**0.022**	0.81	0.52	1.26	0.343	0.89	0.64	1.22	0.451
*TOP2A*	1.19	1.09	1.31	**<0.001**	1.38	1.21	1.56	**<0.001**	1.21	0.96	1.51	0.104	0.99	0.74	1.34	0.971	0.89	0.69	1.15	0.380
*UBE2C*	1.23	1.09	1.39	**0.001**	1.44	1.21	1.71	**<0.001**	1.43	1.01	2.04	**0.046**	0.69	0.45	1.07	0.096	1.17	0.88	1.55	0.277
i-genes																				
*BTN3A2*	0.90	0.77	1.06	0.208	0.87	0.69	1.09	0.220	1.07	0.68	1.68	0.772	0.56	0.35	0.88	**0.013**	1.12	0.81	1.55	0.484
*CCL19*	0.98	0.89	1.09	0.730	0.99	0.86	1.14	0.860	0.89	0.68	1.17	0.403	0.91	0.70	1.18	0.465	1.12	0.89	1.40	0.345
*CD2*	0.96	0.86	1.07	0.445	1.03	0.89	1.20	0.687	0.93	0.70	1.24	0.628	0.61	0.44	0.85	**0.004**	1.06	0.82	1.38	0.650
*CD52*	0.99	0.97	1.01	0.177	0.99	0.96	1.02	0.651	0.98	0.92	1.04	0.467	0.97	0.92	1.02	0.216	0.99	0.95	1.03	0.698
*HLADPA1*	1.00	0.89	1.12	0.973	0.95	0.81	1.11	0.502	0.93	0.69	1.26	0.658	0.89	0.68	1.18	0.426	1.28	0.98	1.67	0.075
*TRBC1*	0.93	0.79	1.09	0.356	0.91	0.72	1.15	0.439	1.01	0.65	1.57	0.960	0.67	0.45	0.99	**0.043**	1.15	0.83	1.60	0.403
Lymph node-negative																				
No. of patients (no. of events)		421 (64)				202 (29)				45 (7)				58 (12)				116 (16)		
p-genes																				
*AURKA*	1.06	0.92	1.21	0.418	1.20	0.98	1.47	0.078	1.01	0.65	1.58	0.963	1.02	0.73	1.43	0.905	0.93	0.72	1.21	0.583
*CCNB2*	1.18	0.91	1.53	0.208	1.37	0.93	2.03	0.114	1.09	0.54	2.22	0.812	0.78	0.37	1.65	0.516	1.34	0.68	2.62	0.398
*FOXM1*	1.34	1.08	1.67	**0.009**	1.57	1.13	2.18	**0.007**	2.22	0.88	5.61	0.093	1.21	0.57	2.54	0.621	1.27	0.73	2.22	0.401
*MKI67*	1.29	1.01	1.64	**0.042**	1.50	1.08	2.08	**0.014**	1.45	0.77	2.74	0.253	1.17	0.65	2.11	0.595	0.89	0.47	1.65	0.702
*MMP11*	1.26	1.06	1.50	**0.008**	1.21	0.95	1.56	0.124	1.98	1.11	3.54	**0.021**	1.56	0.98	2.50	0.063	0.99	0.69	1.42	0.957
*PTTG1*	1.28	0.93	1.77	0.127	1.38	0.86	2.23	0.185	1.34	0.62	2.91	0.455	0.92	0.41	2.06	0.837	1.49	0.74	2.98	0.265
*RACGAP1*	1.16	0.89	1.51	0.264	1.24	0.86	1.78	0.255	1.18	0.47	2.93	0.722	1.46	0.72	2.96	0.291	0.90	0.49	1.65	0.736
*RRM2*	1.35	1.04	1.75	**0.025**	1.74	1.18	2.57	**0.006**	1.43	0.51	3.95	0.495	0.92	0.46	1.84	0.809	1.06	0.62	1.82	0.839
*TOP2A*	1.29	1.09	1.53	**0.004**	1.45	1.14	1.85	**0.002**	1.45	0.85	2.50	0.176	1.17	0.72	1.89	0.527	1.11	0.68	1.80	0.685
*UBE2C*	1.37	1.09	1.73	**0.007**	1.64	1.15	2.32	**0.006**	1.89	0.88	4.06	0.104	0.63	0.31	1.28	0.201	1.53	0.89	2.65	0.125
i-genes																				
*BTN3A2*	0.81	0.60	1.09	0.161	0.65	0.41	1.04	0.074	1.05	0.41	2.71	0.912	0.50	0.26	0.99	**0.045**	1.26	0.75	2.14	0.383
*CCL19*	0.95	0.79	1.14	0.586	0.89	0.68	1.16	0.397	0.70	0.38	1.26	0.234	0.80	0.50	1.27	0.342	1.34	0.93	1.93	0.120
*CD2*	0.88	0.73	1.06	0.182	0.78	0.59	1.05	0.100	0.78	0.39	1.53	0.462	0.60	0.37	0.96	**0.033**	1.31	0.86	2.00	0.207
*CD52*	0.99	0.95	1.03	0.593	0.98	0.92	1.05	0.640	1.00	0.90	1.12	0.986	0.96	0.87	1.05	0.352	1.01	0.95	1.07	0.798
*HLADPA1*	0.93	0.76	1.14	0.484	0.82	0.61	1.10	0.190	0.72	0.37	1.39	0.324	0.80	0.51	1.26	0.344	1.40	0.93	2.11	0.105
*TRBC1*	0.74	0.55	1.00	0.050	0.73	0.45	1.18	0.199	0.61	0.21	1.76	0.362	0.37	0.19	0.74	**0.005**	1.32	0.79	2.20	0.295
Lymph node-positive																				
No. of patients (no. of events)		394 (145)				206 (79)				66 (25)				46 (16)				76 (25)		
p-genes																				
*AURKA*	1.05	0.97	1.15	0.234	1.10	0.97	1.25	0.133	0.99	0.80	1.22	0.922	1.34	1.05	1.72	**0.018**	0.85	0.69	1.05	0.140
*CCNB2*	1.13	0.97	1.32	0.126	1.33	1.07	1.65	**0.010**	1.12	0.74	1.68	0.594	1.08	0.65	1.80	0.771	0.74	0.47	1.15	0.179
*FOXM1*	1.16	0.99	1.37	0.068	1.26	1.01	1.58	**0.038**	1.09	0.69	1.72	0.702	0.95	0.46	2.00	0.901	1.11	0.72	1.69	0.637
*MKI67*	1.26	1.05	1.51	**0.013**	1.51	1.14	2.01	**0.005**	1.29	0.83	2.03	0.258	1.12	0.68	1.85	0.658	0.96	0.60	1.56	0.883
*MMP11*	1.23	1.09	1.39	**0.001**	1.16	0.99	1.37	0.064	1.25	0.93	1.68	0.146	1.51	1.01	2.25	**0.043**	1.29	0.96	1.75	0.090
*PTTG1*	1.00	0.81	1.22	0.967	1.02	0.77	1.35	0.903	1.37	0.84	2.23	0.213	0.96	0.47	1.97	0.908	0.74	0.42	1.30	0.293
*RACGAP1*	1.15	0.96	1.37	0.137	1.22	0.96	1.54	0.100	1.33	0.79	2.23	0.286	0.83	0.45	1.53	0.551	1.04	0.69	1.56	0.859
*RRM2*	1.14	0.96	1.34	0.128	1.26	1.00	1.58	**0.046**	1.66	1.07	2.58	**0.024**	0.81	0.46	1.41	0.453	0.86	0.61	1.22	0.402
*TOP2A*	1.16	1.05	1.29	**0.005**	1.29	1.11	1.50	**0.001**	1.20	0.94	1.54	0.152	0.91	0.63	1.30	0.594	0.92	0.69	1.24	0.591
*UBE2C*	1.18	1.02	1.36	**0.023**	1.32	1.08	1.60	**0.006**	1.30	0.88	1.94	0.192	0.81	0.46	1.40	0.442	1.04	0.77	1.40	0.802
i-genes																				
*BTN3A2*	1.01	0.84	1.22	0.905	1.05	0.81	1.38	0.702	1.15	0.68	1.94	0.609	0.65	0.34	1.25	0.193	1.06	0.72	1.57	0.769
*CCL19*	0.98	0.87	1.11	0.756	1.02	0.86	1.20	0.856	0.94	0.70	1.27	0.682	0.93	0.68	1.28	0.670	0.98	0.73	1.31	0.884
*CD2*	1.00	0.87	1.15	0.958	1.15	0.95	1.38	0.150	0.93	0.68	1.27	0.658	0.64	0.40	1.02	0.063	0.93	0.67	1.31	0.689
*CD52*	0.98	0.96	1.01	0.167	0.99	0.96	1.03	0.646	0.97	0.90	1.05	0.470	0.97	0.91	1.04	0.395	0.98	0.93	1.04	0.478
*HLADPA1*	1.02	0.89	1.17	0.767	0.98	0.82	1.17	0.811	1.03	0.72	1.45	0.885	0.93	0.65	1.33	0.688	1.26	0.86	1.85	0.242
*TRBC1*	0.99	0.81	1.20	0.885	0.96	0.73	1.26	0.756	1.20	0.74	1.93	0.463	0.90	0.52	1.57	0.710	1.01	0.66	1.56	0.948

Hazard ratios with *P* values < 0.05 are marked in bold

*HR* hormone receptor, *HER2* human epidermal growth factor receptor 2, *TNBC* triple-negative breast cancer, *CI* confidence interval, *p-genes* proliferation-related genes, *i-genes* immune response-related genes

Subgroup analysis by LN status in each molecular subtype showed a slight difference in the significant genes between LN+ and LN**−** cancers. In particular, in HR+/HER2**−**, LN**−** tumors, five p-genes (*FOXM1*, *MK167*, *RRM2*, *TOP2A*, and *UBE2C*) were statistically significant and the expression of immune response-related *BTN3A2* showed a marginal significance in DMFS (Table 3). However, *BTN3A2* was not significant in LN+ breast cancer. Three p-genes (*MMP11, RRM2*, and *UBE2C*) were significant in DMFS in the HR+/HER2+ subtype. In HR**−**/HER2+ breast cancer, *MMP11* and three i-genes (*BTN3A2, CD2*, and *TRBC1*) were significantly associated with clinical outcomes, whereas no clinical variable was significant. Higher *MMP11* expression was significantly associated with higher risk of distant metastasis, while higher expression levels of *BTN3A2*, *CD2*, and *TRBC1* were related to favorable outcomes (Table 3). The significant association of these i-genes with a favorable outcome was only observed in LN**−** breast cancer, and not in LN+ tumors.

Consistent with the findings for DMFS, genes significantly associated with DFS or OS were dependent on molecular subtype and the list of genes was similar to that associated with DMFS (Supplementary Tables S2, S3). A significant relationship between high level expression of i-genes and favorable outcome was also observed only in HR**−**/HER2+ breast cancer. Subgroup analysis by LN status in each molecular subtype showed only a slight difference between LN**−** and LN+ breast cancer in the list of genes significantly associated with DFS or OS.

### Multivariate analysis according to molecular subtype

Using the clinical and gene variables that were significant in the univariate analysis, stepwise variable selection of multivariate analysis was performed to identify independent predictors of DMFS for each molecular subtype. Hazard ratios and 95% confidence intervals (CIs) for DMFS are shown in Table 4. In HR**−**/HER2+ breast cancer, *MMP11* (hazard ratio 1.49; 95% CI 1.08–2.04; *P* = 0.014) and *CD2* (hazard ratio 0.66; 95% CI 0.47–0.94; *P* = 0.022) retained their statistical significance for DMFS in multivariate analysis. These results demonstrated that the expression of *MMP11* and *CD2* are independent prognostic factors for HR**−**/HER2+ breast cancer. In other subtypes, positive LN status was an independent negative prognostic factor. Moreover, *TOP2A* was independently associated with DMFS in the HR+/HER2**−** subtype.

**Table 4:** Multivariate analysis of DMFS according to molecular subtype

	All				HR+/HER2−				HR+/HER2+				HR−/HER2+				TNBC			
	Hazard ratio	95% CI		*P* value	Hazard ratio	95% CI		*P* value	Hazard ratio	95% CI		*P* value	Hazard ratio	95% CI		*P* value	Hazard ratio	95% CI		*P* value
No. of patients (no. of events)		815 (209)				408 (108)				111 (32)				104 (28)				192 (41)		
Lymph node status																				
Negative	1.00				1.00				1.00								1.00			
Positive	2.74	2.03	3.68	**<0.001**	2.95	1.91	4.56	**<0.001**	2.75	1.19	6.38	**0.018**					2.82	1.51	5.29	**0.001**
p-genes																				
*MKI67*	1.13	0.96	1.32	0.149	1.25	0.99	1.58	0.059												
*MMP11*	1.22	1.11	1.35	**<0.001**	1.12	0.98	1.29	0.104	1.32	1.00	1.73	0.050	1.49	1.08	2.04	**0.014**				
*RRM2*									1.48	0.97	2.28	0.070								
*TOP2A*	1.16	1.05	1.28	**0.004**	1.26	1.09	1.44	**0.001**												
i-genes																				
*CD2*													0.66	0.47	0.94	**0.022**				

Hazard ratios with *P* values < 0.05 are marked in bold

*HR* hormone receptor, *HER2* human epidermal growth factor receptor 2, *TNBC* triple-negative breast cancer, *CI* confidence interval, *p-genes* proliferation-related genes, *i-genes* immune response-related genes

With regard to DFS, the expression of *MMP11* and *UBE2C* were independent prognostic factors in HR**−**/HER2+ breast cancer (Supplementary Table S4). Unexpectedly, increased *UBE2C* expression showed a significant association with the decreased risk of recurrence in this subtype. In HR+/HER2**−** cancers, LN+ status and expression of *MMP11* and *TOP2A* were independently associated with the increased risk of recurrence. Age, LN status, and *UBE2C* expression were independent prognostic factors in the HR+/HER2+ cancer. LN status was an independent negative prognostic factor only in TNBC.

Independent prognostic factors for OS in HR**−**/HER2+ breast cancer included the expression of *MMP11* and *BTN3A2* (Supplementary Table S4). In the HR+/HER2+ subtype, *RRM2* expression retained its significance. By contrast, LN status only was an independent prognostic for OS in HER2**−** breast cancer, whereas no gene variable was significant.

### Prognostic performance of the risk model for distant metastasis in HR−/HER2+ breast cancer

**Fig. 1:**
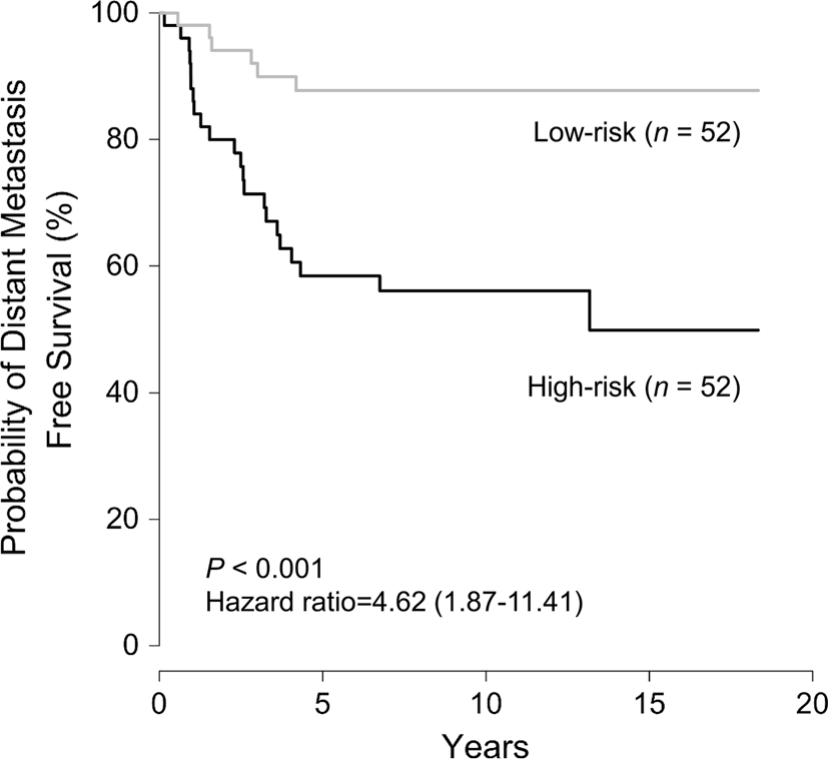
Kaplan–Meier plot of distant metastasis-free survival (DMFS) in low-risk and high-risk groups defined by our prognostic model in patients with HR**−**/HER2+ breast cancer. Survival estimates between two risk groups were compared using the log-rank test and the hazard ratio was derived using Cox proportional hazard model

To assess the prognostic significance of our prognostic model, HR**−**/HER2+ breast cancer patients were classified into two groups, high risk and low risk, according to the risk score developed by our prognostic model. Kaplan–Meier curves demonstrated that DMFS for patients in the high-risk group was significantly lower than for those in the low-risk group (log-rank test; *P* < 0.001; Fig. 1). The probabilities of DMFS at 10 years for patients in the high-risk and low-risk groups were 56.1% and 87.7%, respectively. That is, patients in the high-risk group had a significantly higher 10-year distant metastasis rate (43.9%) than those in the low-risk group (12.3%). When we analyzed the difference in clinical characteristics between the risk groups, we found no significant differences between the two groups (Supplementary Table S5). These results indicate that our prognostic model is useful for differentiating HR**−**/HER2+ breast cancer patients at high risk and low risk of distant metastasis, whereas clinical variables alone are not sufficient to identify these patients. There was no association between clinical variables with our risk score in HR**−**/HER2+ breast cancer (Supplementary Fig. S2).

In the multivariate analysis after adjustments for traditional clinicopathological parameters, our risk score retained statistical significance (hazard ratio 2.49; 95% CI 1.46–4.24; *P* = 0.001; Table 5) and was more significant than other prognostic models based on clinical variables (Table 6). These results indicate that our model is an independent prognostic indicator of risk of distant metastasis in HR**−**/HER2+ breast cancer.

**Table 5:** Multivariate analysis of our prognostic model and traditional clinicopathological parameters for DMFS in HR−/HER2+ breast cancer

	Univariate analysis			Multivariate analysis		
	Hazard ratio	95% CI	*P* value	Hazard ratio	95% CI	*P* value
No. of patients (no. of events)		104 (28)			104 (28)	
Risk score	2.36	(1.48–3.78)	**<0.001**	2.49	(1.46–4.24)	**0.001**
Histologic grade						
1 and 2	1.00			1.00		
3	1.27	(0.50–3.21)	0.618	1.79	(0.69–4.65)	0.229
Lymph node status						
Negative	1.00			1.00		
Positive	1.87	(0.88–3.96)	0.101	1.67	(0.71–3.91)	0.238
Tumor size (cm)			0.211			0.155
≤2	1.00			1.00		
2–5	2.39	(0.90–6.35)	0.081	2.85	(0.94–8.61)	0.063
>5	1.68	(0.32–8.64)	0.538	1.44	(0.25–8.35)	0.684

Hazard ratios with *P* values < 0.05 are marked in bold

*CI* confidence interval

**Table 6:** Multivariate analysis of our prognostic model and other prognostic models based on traditional clinicopathological parameters for DMFS in HR−/HER2+ breast cancer

	Univariate analysis			Multivariate analysis		
	Hazard ratio	95% CI	*P* value	Hazard ratio	95% CI	*P* value
No. of patients (No. of events)		104 (28)			104 (28)	
Risk score	2.36	(1.48–3.78)	**<0.001**	2.49	(1.46–4.24)	**0.001**
PREDICT	1.03	(1.02–1.05)	**<0.001**	1.02	(1.00–1.05)	0.082
SNAP	1.04	(1.02–1.07)	**0.001**	1.06	(1.01–1.11)	**0.021**
NPI score	1.56	(1.15–2.14)	**0.005**	2.06	(1.07–3.97)	**0.031**

Hazard ratios with *P* values < 0.05 are marked in bold

*CI* confidence interval, *PREDICT* www.predict.nhs.uk, *SNAP* www.CancerMath.net, *NPI* Nottingham prognostic index

**Fig. 2:**
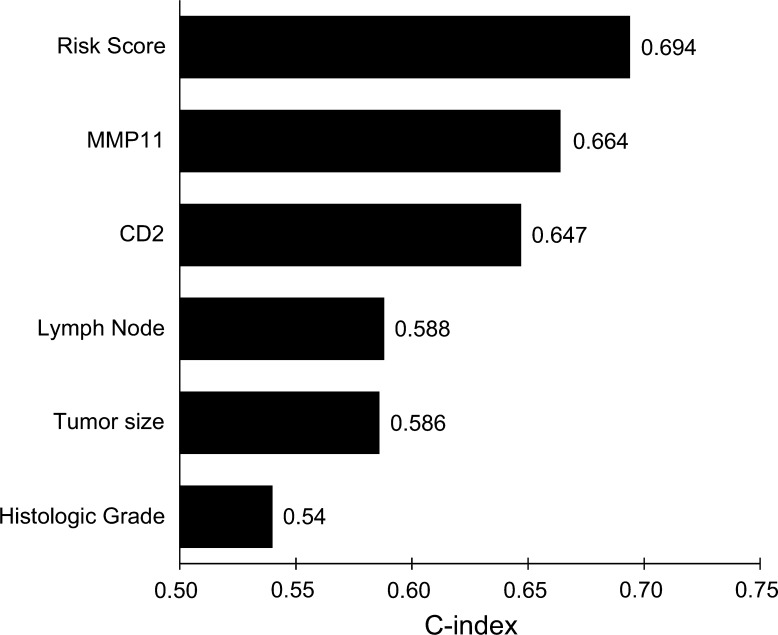
Prognostic performance of our risk score in predicting distant metastasis in HR**−**/HER2+ breast cancer compared with that of traditional clinicopathological parameters based on C-index. Values on the *x-axis* are unbiased estimates of the C-index of the linear combination of one or more variables by Cox regression

Our model showed the best performance in predicting the risk of distant metastasis with the highest C-index (0.694) among other traditional prognostic factors (Fig. 2) or prognostic models based on clinicopathological factors alone (Supplementary Fig. S3). These results reinforce that our prognostic model is superior to other conventional models based on clinical variables alone in predicting the risk of distant metastasis in HR**−**/HER2+ breast cancer and provides more accurate prognostic information than traditional clinicopathological factors in this subtype of breast cancer.

### Prognostic significance of *MMP11* and *CD2* expression in HR−/HER2+ breast cancer in public dataset

**Fig. 3:**
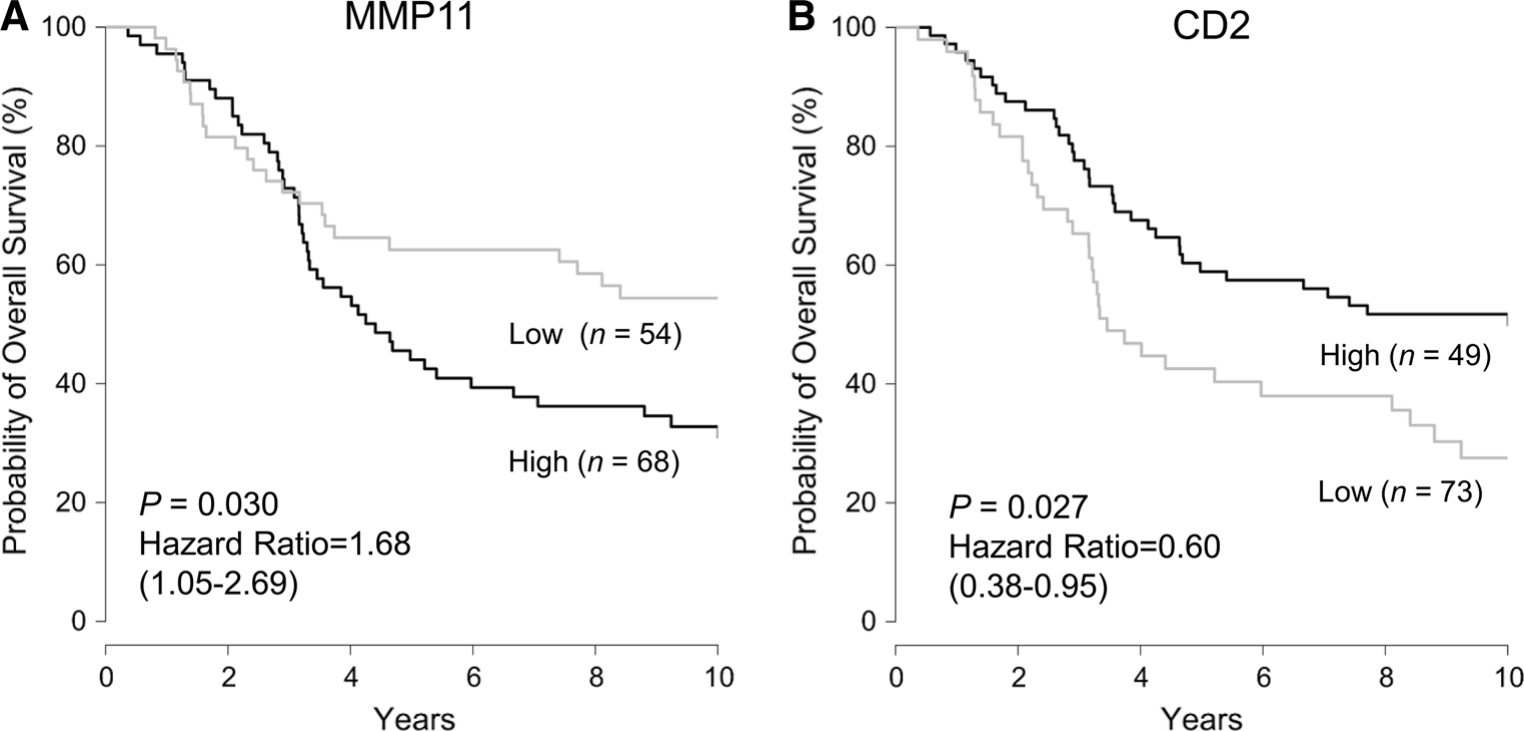
Prognostic significance of *MMP11* and *CD2* gene expression in public dataset. Kaplan–Meier plot of overall survival in two subgroups classified based on the expression of **a** *MMP11* or **b** *CD2* in patients with HR−/HER2+ breast cancer from Molecular Taxonomy of Breast Cancer International Consortium (METABRIC) cohort

We also examined the relationship between *MMP11* and *CD2* gene expression and prognosis of patients with HR**−**/HER2+ breast cancer using public dataset to confirm their clinical significance in other cohorts. Gene expression and clinical data from METABRIC (Molecular Taxonomy of Breast Cancer International Consortium) cohort [28] were obtained from cBioPortal (http://www.cbioportal.org/) [29]. Consistent with the results in our cohort, significantly shorter OS in patients with high *MMP11* expression than those with low *MMP11* expression was observed (*P* = 0.030), whereas patients with high *CD2* expression had a significantly longer OS than those with low *CD2* expression (*P* = 0.027) (Fig. 3).

## Discussion

Based on the 384 genes identified from our previous study, we selected 16 candidate prognostic genes, and assessed the association between their expression and patient outcome in different molecular subtypes of breast cancer.

Univariate analysis identified significant factors correlated with distant metastasis in different molecular subtypes of breast cancer. Among the traditional clinicopathological factors, LN status showed a significant relationship with DMFS and DFS in all molecular subtypes except HR**−**/HER2+ breast cancer. Of note, larger tumor size was significantly associated with higher risk of distant metastasis in HER2**−** breast cancer, but not HER2+ breast cancer. Moreover, we identified subtype-specific prognostic genes whose expression was significantly associated with the risk of distant metastasis. Higher expression of most of the p-genes correlated significantly with a higher risk of distant metastasis in HR+/HER2**−** cancer, whereas no significance was observed in TNBC. These results are consistent with the previous finding that proliferation is the most important component of the prognostic signature in ER+ breast cancer [12]. Our study provides novel proliferation-related prognostic gene sets for HR+ breast cancer that may be used to develop a multigene assay for predicting the risk of distant recurrence and thereby identify patients who will benefit from specific treatment in this subtype.

In addition, elevated expression of i-genes (*BTN3A2*, *CD2*, and *TRBC1*) was significantly correlated with favorable clinical outcome in HR**−**/HER2+ breast cancer, but not in other subtypes. The prognostic value of immune gene signatures as predictors of distant metastasis in HR**−** breast cancer has been reported [13, 14, 16]. In particular, both *BTN3A2* and *CD2* are involved in the T-cell immune response. However, it is difficult to exclude the possibility that the increased expression of these genes is due to infiltrating immune cells. The prognostic significance of infiltrating immune cells as a key component of the tumor microenvironment has been recognized for breast cancer, and a positive correlation between lymphocyte infiltration or expression of lymphocyte-associated genes and HER2 amplification/overexpression in breast cancer has been reported [30, 31]. Moreover, higher expression of lymphocyte-associated genes is associated with a favorable prognosis in HER2+ breast cancer [30–32]. Our findings further expand the prognostic significance of i-genes in HR−/HER2+ breast cancer. It is notable that among i-genes, T-cell-related genes are associated with favorable prognosis of HR**−**/HER2+ breast cancer in our study. This is supported by a recent study reporting that T-cells, but not B-cells, have significant prognostic value in HER2+ breast cancer [32].

Interestingly, a recent study revealed that CD2 is critical for antibody-dependent responses by adaptive natural killer (NK) cells, suggesting an important role for CD2 in stimulating the NK cell response to therapeutic antibodies [33]. This recent finding raises the possibility that the correlation between high *CD2* expression and favorable prognosis of patients with HR**−**/HER2+ breast cancer in our study is related to the augmentation of NK cytotoxic activity against cancer cells by CD2. However, this relationship was not assessed in this study, and further studies designed to unravel the association between *CD2* expression and anti-HER2 antibody response or the value of *CD2* in predicting anti-HER2 antibody response in HER2+ breast cancer will be required.

Importantly, we found that the gene expression of *MMP11* and *CD2* are independent prognostic factors for DMFS in HR**−**/HER2+ breast cancer, whereas clinical variables were not significant prognostic indicators. With regard to prognostic models for HR**−**/HER2+ breast cancer, several attempts have been made to identify prognostic multigene signatures for this subtype using gene expression microarray data, but a few validated prognostic genes have been established. In this context, it is important that the expression of *MMP11* and *CD2* are validated as independent prognostic factors and this is in line with previous studies showing that the main gene signatures associated with prognosis in HER2+ breast cancer include genes related to tumor invasion and immune response [12, 34]. The roles of MMP11 in tumor progression have been reported in breast cancer. Its overexpression promotes anoikis resistance [35] and enhances tumorigenesis in HER2**−** breast cancer cell lines via IGF-1 signaling [36]. Recent studies also showed that MMP11 is a downstream target of oncogene or tumor suppressor microRNA, thereby contributes to tumor cell migration, invasion, or angiogenesis in breast cancer cells. Oncogenic transcription factor Gli1 promotes migration and invasion of ER− breast cancer cells through the up-regulation of *MMP11* [37] and reduced MMP11 expression mediates the anti-angiogenic and invasion effect of microRNA miR-98 in ER− breast cancer cells [38]. However, the clinical and functional significance of MMP11 in HR**−**/HER2+ breast cancer remains unclear. Here, our findings demonstrate for the first time the prognostic significance of *MMP11* and *CD2* expression in HR**−**/HER2+ breast cancer and suggest that they are promising biomarkers or drug targets for this subtype of breast cancer. Further studies for validation will be required.

Generally, patients with ER**−** breast cancer have a worse prognosis than those with ER+ breast cancer [39–41]. In contrast, there were no statistically significant differences in patient survivals between molecular subtypes during the entire follow-up period in our study. This discrepancy may be in part due to the chemotherapy effects on the subtypes. Most patients (86.3%) including TNBC patients (92.7%) of our study received adjuvant chemotherapy and our previous study [19] demonstrated that TNBC patients with chemotherapy had significantly longer DFS and OS than those without chemotherapy, whereas TNBC without chemotherapy showed a relatively worse prognosis. However, a significant population of ER**−** breast cancer cases not receiving adjuvant chemotherapy has a good prognosis [34]. More accurately identifying these patients is important because this population may benefit from less aggressive therapy. Our data revealed a significant difference in DMFS between high-risk and low-risk groups as defined by our prognostic model, illustrating that our model can discriminate patients at low risk and high risk of distant metastasis in HR**−**/HER2+ breast cancer. Therefore, our prognostic model may help to guide treatment for patients with HR**−**/HER2+ breast cancer by identifying those with a good prognosis within this subtype.

## Conclusions

In summary, we identified molecular subtype-specific novel prognostic genes in breast cancer and developed a novel prognostic model to predict the risk of distant metastasis for HR**−**/HER2+ breast cancer based on the gene expression of *MMP11* and *CD2*. Our prognostic model was superior to traditional clinicopathological factors in prognostic performance and may be used in identifying patients with good prognosis from this aggressive subtype of breast cancer. Consequently, the novel prognostic genes validated in this study may be used to develop assays to accurately predict the prognosis of these patients and thereby provide useful information for determining treatment options in patients with HR**−**/HER2+ breast cancer.

## Electronic supplementary material

Below is the link to the electronic supplementary material.

Supplementary material 1 (PDF 1026 kb)

## Abbreviations

C-index: Harrell’s concordance index
CI: Confidence interval
DFS: Disease-free survival
DMFS: Distant metastasis-free survival
ER: Estrogen receptor
FFPE: Formalin-fixed, paraffin-embedded
HER2: Human epidermal growth factor receptor 2
HR: Hormone receptor
IRB: Institutional review board
NPI: Nottingham prognostic index
OS: Overall survival
PR: Progesterone receptor
qRT-PCR: Quantitative real-time reverse transcription-PCR
TNBC: Triple-negative breast cancer
