# Supplementary material for: MMP11 and CD2 as novel prognostic factors in hormone receptor-negative, HER2-positive breast cancer
Source: Breast Cancer Res Treat. 2017 Apr 13;164(1):41–56. doi: 10.1007/s10549-017-4234-4 (PMC5487710; doi:10.1007/s10549-017-4234-4)
Supplement: Supplementary file 1 — Supplementary material 1 (PDF 1026 kb) [file 10549_2017_4234_MOESM1_ESM.pdf]

MMP11 and CD2 as Novel Prognostic Factors in Hormone Receptor-Negative, HER2-Positive Breast Cancer

Jinil Han, Yoon-La Choi, Haein Kim, Jun Young Choi, Se Kyung Lee, Jeong Eon Lee, Joon-Seok Choi, Sarah Park, Jong-Sun Choi,Young Deug Kim, Seok Jin Nam, Byung-Ho Nam, Mi Jeong Kwon*, Young Kee Shin*

*Corresponding authors: Mi Jeong Kwon (mjkwon94@knu.ac.kr) and Young Kee Shin (ykeeshin@snu.ac.kr)

Supplementary materials and methods

Study population

A total of 997 FFPE tissue specimens were obtained from patients with breast cancer who underwent curative resection for primary tumors with LN dissection at the SMC between October 1994 and December 2002. Patients with stage I–III cancer were included, and had undergone either mastectomy or breast-conserving surgery followed by radiotherapy, chemotherapy, or hormone therapy, either alone or in combination, according to conventional regimens. No patients included in this study received anti-HER2 therapy. We also obtained 50 frozen tissue samples paired with FFPE samples from the same patients. We excluded FFPE samples in which more than 50% of the area had been infiltrated by inflammatory cells or showed interstitial fibrosis or fat. We also excluded samples in which the duct and lobule tissue represented less than 30% of the total sample area. Each patient's medical records were reviewed for clinicopathological information, including patient age, tumor size, LN status, pathologic stage, type of treatment and treatment outcome.

Selection of candidate prognostic genes

A set of 384 candidate genes associated with patient prognosis in early breast cancer were identified in our previous study using public gene expression microarray data [1]. As described previously [1], two major factors accounting for the expression patterns of the selected 384 genes were found using principal component analysis and their biological functions were investigated using gene ontology (GO) analysis. As a result, the first principal component was involved in proliferation activity, whereas the second principal component was related to immune response. Therefore, these 384 candidate genes were classified into two categories according to their biological functions: proliferation-related genes (p-genes)

and immune response-related genes (i-genes). From these candidate genes, a total of 30 candidate genes were selected based on the following criteria: (1) high correlation with either proliferation or immune response, (2) high variability between samples (large interquartile range), and (3) high mean expression value. Based on the results of qRT-PCR, 16 genes with high correlation of expression between FFPE and frozen tissues were further selected. These 16 genes included 10 p-genes (*AURKA*, *CCNB2*, *FOXM1*, *MKI67*, *MMP11*, *PTTG1*, *RACGAP1*, *RRM2*, *TOP2A*, and *UBE2C*) and six i-genes (*BTN3A2*, *CCL19*, *CD2*, *CD52*, *HLA.DPA1*, and *TRBC1*).

qRT-PCR

RNA was extracted from frozen tissues using an RNeasy mini kit (Qiagen, Hilden, Germany) and from FFPE tissue samples using the Arcturus kit (Life Technologies, Grand Island, NY, USA) or Tissue Preparation System (Siemens AG, Munich, Germany). qRT-PCR was carried out using the LightCycler 480 system (Roche Applied Science, Mannheim, Germany) with a QuantiFast Multiplex RT-PCR Kit (Qiagen) according to the manufacturer's instructions. Reagents were dispensed into 384-well plates using an automated dispenser (STARlet; Hamilton Robotics, Reno, NV, USA).

Normalization of qRT-PCR data

To select suitable references genes for the normalization of qRT-PCR expression of the 16 candidate genes, the expression levels of five candidate reference genes were also measured. These genes included three novel reference genes (*UBQLN1*, *CUL1*, and *CTBP1*) identified in our previous study [2] and two traditional reference genes (*TBP* and *HMBS*). First, the expression levels of these five genes were measured together with those of the 16 candidate prognostic genes by qRT-PCR in paired FFPE and frozen samples. Of the five candidate

genes, three genes (*CTBP1*, *CUL1*, and *UBQLN1*) showed relatively similar expression levels compared with the expression levels of the 16 genes, and there was good correlation between the frozen and FFPE samples (data not shown). When measured in the 926 FFPE samples, these genes also had relatively fewer missing Cq values (25 missing values for *CTBP1*, 89 missing values for *CUL1*, and 15 missing values for *UBQLN1*) than the other two genes (251 missing values for *HMBS* and 128 missing values for *TBP*). They also showed a high correlation in gene expression with each other (Pearson coefficient > 0.79; data not shown). Additionally, analyses using geNorm [3] and NormFinder [4] algorithms implemented in the R package NormqPCR ranked *CTBP1*, *CUL1*, and *UBQLN1* as the top three stable genes (data not shown). Therefore, these three genes were selected as reference genes for normalization of the expression of the 16 candidate prognostic genes.

Development of the prognostic model for HR−/HER2+ breast cancer and cross validation

Based on stepwise multivariate analyses results, a prognostic model or algorithm to predict the risk of distant metastasis in HR−/HER2+ breast cancer was developed. Relative expression values of the two prognostic genes normalized by the average expression level of the three reference genes were used to calculate the risk score, a molecular predictor of distant metastasis. The coefficient values for each variable were obtained from the Cox's proportional hazard model and the risk score was defined as a linear combination of the coefficients to predict distant metastasis in HR−/HER2+ breast cancer as follows:

$$\text{Risk score} = 0.45 \times \triangle C_q\_\textit{MMP11} - 0.48 \times \triangle C_q\_\textit{CD2}.$$

Higher values indicate a higher risk of distant metastasis. A cutoff risk score value to distinguish patients with low- and high-risk for distant metastasis was set at -0.53, which

maximized the sum of sensitivity and specificity. A patient was assigned to the ‘high-risk’ group if the risk score of the sample was ≥ -0.53. Otherwise, the patient was considered to belong to the ‘low-risk’ group. For development and performance evaluation of the prognostic model, a 10 fold-cross validation procedure was used [5].

The Nottingham Prognostic Index (NPI) score was used to calculate risk based on clinicopathological factors alone [6]. The NPI score was calculated as follows: 0.2×tumor size (cm) + tumor grade + nodal status. This NPI prognostic value was calculated for each of the samples, which were classified into four prognostic groups based on the NPI score: excellent, 2–2.4; good, 2.4–3.4; moderate, 3.4–5.4; and poor, >5.4. We also compared the prognostic value or performance of our prognostic model with other prognostic models based on clinical variables, including two web-based prediction tools, SNAP (www.CancerMath.net) [7] and PREDICT (www.predict.nhs.uk) [8,9]. In SNAP, survival rates are calculated using age, tumor size, nodal status, ER/PR/HER2 status, tumor grade and histologic type. Similarly, PREDICT is a breast cancer prognostication and treatment benefit tool based on prognostic factors such as age, tumor size, tumor grade, number of positive LNs, and ER/HER2/Ki-67 status. The Harrell’s concordance index (C-index) [10] was calculated to estimate the discrimination capability of each prognostic model and to compare their prognostic performance.

References

1. Oh E, Choi YL, Park T, Lee S, Nam SJ, Shin YK (2012) A prognostic model for lymph node-negative breast cancer patients based on the integration of proliferation and immunity. Breast cancer research and treatment 132 (2):499-509. doi:10.1007/s10549-011-1626-8
2. Kwon MJ, Oh E, Lee S, Roh MR, Kim SE, Lee Y, Choi YL, In YH, Park T, Koh SS, Shin YK (2009) Identification of novel reference genes using multiplatform expression data and their validation for quantitative gene expression analysis. PloS one 4 (7):e6162. doi:10.1371/journal.pone.0006162
3. Vandesompele J, De Preter K, Pattyn F, Poppe B, Van Roy N, De Paepe A, Speleman F (2002) Accurate normalization of real-time quantitative RT-PCR data by geometric averaging of multiple internal control genes. Genome biology 3 (7):RESEARCH0034
4. Andersen CL, Jensen JL, Orntoft TF (2004) Normalization of real-time quantitative reverse transcription-PCR data: a model-based variance estimation approach to identify genes suited for normalization, applied to bladder and colon cancer data sets. Cancer research 64 (15):5245-5250. doi:10.1158/0008-5472.
5. Tournoud M, Larue A, Cazalis MA, Venet F, Pachot A, Monneret G, Lepape A, Veyrieras JB (2015) A strategy to build and validate a prognostic biomarker model based on RT-qPCR gene expression and clinical covariates. BMC Bioinformatics 16:106. doi:10.1186/s12859-015-0537-9
6. Haybittle JL, Blamey RW, Elston CW, Johnson J, Doyle PJ, Campbell FC, Nicholson RI, Griffiths K (1982) A prognostic index in primary breast cancer. Br J Cancer 45 (3):361-366
7. Chen LL, Nolan ME, Silverstein MJ, Mihm MC, Jr., Sober AJ, Tanabe KK, Smith BL, Younger J, Michaelson JS (2009) The impact of primary tumor size, lymph node status, and other prognostic factors on the risk of cancer death. Cancer 115 (21):5071-5083. doi:10.1002/cncr.24565
8. Wishart GC, Azzato EM, Greenberg DC, Rashbass J, Kearins O, Lawrence G, Caldas C, Pharoah PD (2010) PREDICT: a new UK prognostic model that predicts survival following surgery for invasive breast cancer. Breast Cancer Res 12 (1):R1. doi:10.1186/bcr2464
9. Wishart GC, Bajdik CD, Dicks E, Provenzano E, Schmidt MK, Sherman M, Greenberg DC, Green AR, Gelmon KA, Kosma VM, Olson JE, Beckmann MW, Winqvist R, Cross SS, Severi G, Huntsman D, Pylkas K, Ellis I, Nielsen TO, Giles G, Blomqvist C, Fasching PA, Couch FJ, Rakha E, Foulkes WD, Blows FM, Begin LR, van't Veer LJ, Southey M, Nevanlinna H, Mannermaa A, Cox A, Cheang M, Baglietto L, Caldas C, Garcia-Closas M, Pharoah PD (2012) PREDICT Plus: development and validation of a prognostic model for early breast cancer that includes HER2. Br J Cancer 107 (5):800-807. doi:10.1038/bjc.2012.338
10. Harrell FE, Lee KL, Mark DB (1996) Tutorial in biostatistics multivariableprognosticmodels: issues in developing models, evaluating assumptions and adequacy, and measuring and reducing errors. Statistics in medicine 15:361-387

Supplementary Tables

Supplementary Table S1. Univariate analysis of clinical variables for DFS and OS according to molecular subtype

	Total				HR+/HER2−				HR+/HER2+				HR−/HER2+				TNBC			
	**Hazard ratio**	**95% CI**		***P* value**	**Hazard ratio**	**95% CI**		***P* value**	**Hazard ratio**	**95% CI**		***P* value**	**Hazard ratio**	**95% CI**		***P* value**	**Hazard ratio**	**95% CI**		***P* value**
**DFS**																				
**No. of patients (No. of events)**	817 (233)				410 (120)				111(37)				104 (33)				192(43)			
**Age (years)**																				
<50	1.00				1.00				1.00				1.00				1.00			
≥50	0.72	0.55	0.94	**0.017**	0.77	0.53	1.13	0.181	0.39	0.16	0.94	**0.036**	0.75	0.38	1.49	0.411	0.71	0.36	1.38	0.309
**Tumor size (cm)**																				
≤2	1.00				1.00				1.00				1.00				1.00			
2-5	1.63	1.22	2.16	**0.001**	1.63	1.11	2.40	**0.013**	0.98	0.49	1.96	0.960	1.98	0.85	4.61	0.112	2.04	0.99	4.20	0.053
>5	1.84	1.10	3.09	**0.021**	1.99	0.97	4.11	0.061	0.88	0.20	3.92	0.868	1.24	0.26	5.95	0.792	3.66	1.25	10.74	**0.018**
**Lymph node status**																				
Negative	1.00				1.00				1.00				1.00				1.00			
Positive	2.44	1.86	3.21	**<0.001**	2.67	1.80	3.96	**<0.001**	2.98	1.36	6.51	**0.006**	1.48	0.75	2.94	0.259	2.50	1.37	4.59	**0.003**
**Histologic grade**																				
1	1.00				1.00				1.00				1.00				-			
2	1.60	0.93	2.73	0.088	1.64	0.89	3.00	0.110	0.82	0.17	3.97	0.809	2.16	0.27	17.09	0.466	1.00			
3	2.08	1.23	3.51	**0.006**	2.31	1.24	4.28	**0.008**	2.33	0.55	9.87	0.251	1.46	0.19	10.96	0.712	1.22	0.56	2.70	0.616
**OS**																				
**No. of patients (No. of events)**	815 (195)				410 (94)				110 (31)				105 (27)				190 (43)			
**Age (years)**																				
<50	1.00				1.00				1.00				1.00				1.00			
≥50	0.95	0.71	1.27	0.721	1.09	0.72	1.65	0.679	0.81	0.36	1.81	0.601	0.68	0.32	1.44	0.311	0.97	0.52	1.83	0.937
**Tumor size (cm)**																				
≤2	1.00				1.00				1.00				1.00				1.00			
2-5	1.63	1.19	2.24	**0.003**	1.58	1.02	2.44	**0.041**	1.11	0.50	2.48	0.793	1.72	0.69	4.31	0.247	2.01	0.94	4.29	0.071
>5	2.71	1.65	4.44	**<0.001**	2.22	1.02	4.81	**0.044**	2.99	0.92	9.75	0.070	1.23	0.25	6.07	0.804	6.15	2.37	15.95	**<0.001**
**Lymph node status**																				
Negative	1.00				1.00				1.00				1.00				1.00			
Positive	2.54	1.88	3.44	**<0.001**	3.08	1.94	4.90	**<0.001**	1.87	0.86	4.08	0.113	1.45	0.68	3.09	0.334	3.44	1.84	6.45	**<0.001**
**Histologic grade**																				
1	1.00				1.00				1.00				-				-			
2	2.03	1.04	3.95	**0.038**	1.66	0.81	3.43	0.168	1.41	0.17	11.74	0.749	1.00				1.00			
3	2.97	1.55	5.68	**0.001**	2.91	1.41	5.99	**0.004**	4.34	0.57	33.18	0.158	0.62	0.27	1.42	0.260	1.15	0.52	2.53	0.729

Abbreviations: HR, hormone receptor; HER2, human epidermal growth factor receptor 2; TNBC, triple-negative breast cancer; CI, confidence interval. Hazard ratios with *P* values < 0.05 are marked in bold.

Supplementary Table S2. Univariate analysis of gene variables for DFS according to molecular subtype

	All				HR+/HER2−				HR+/HER2+				HR−/HER2+				TNBC			
	Hazard ratio	95% CI		*P* value	Hazard ratio	95% CI		*P* value	Hazard ratio	95% CI		*P* value	Hazard ratio	95% CI		*P* value	Hazard ratio	95% CI		*P* value
**No. of patients (No. of events)**	817 (233)				410 (120)				111(37)				104 (33)				192(43)			
**p-genes**																				
AURKA	1.07	1.00	1.15	**0.049**	1.17	1.06	1.29	**0.002**	1.02	0.85	1.21	0.869	1.18	0.98	1.42	0.078	0.90	0.77	1.06	0.207
CCNB2	1.13	0.99	1.28	0.063	1.38	1.15	1.65	**0.001**	1.05	0.76	1.44	0.766	0.99	0.66	1.49	0.959	0.91	0.63	1.30	0.592
FOXM1	1.16	1.03	1.30	**0.017**	1.41	1.19	1.67	**<0.001**	1.17	0.81	1.69	0.401	0.95	0.58	1.56	0.846	1.01	0.73	1.40	0.937
MKI67	1.21	1.06	1.38	**0.006**	1.40	1.16	1.69	**0.001**	1.20	0.87	1.65	0.277	1.14	0.80	1.63	0.456	0.99	0.68	1.43	0.952
MMP11	1.28	1.17	1.41	**<0.001**	1.27	1.12	1.45	**<0.001**	1.30	1.03	1.64	**0.029**	1.45	1.11	1.91	**0.007**	1.17	0.93	1.46	0.178
PTTG1	1.01	0.86	1.19	0.887	1.05	0.82	1.33	0.708	1.23	0.85	1.77	0.264	0.93	0.57	1.52	0.761	0.89	0.58	1.36	0.579
RACGAP1	1.12	0.98	1.29	0.102	1.24	1.03	1.49	**0.022**	1.12	0.74	1.69	0.587	1.11	0.74	1.67	0.620	0.96	0.68	1.36	0.830
RRM2	1.17	1.02	1.34	**0.021**	1.46	1.21	1.77	**<0.001**	1.39	0.93	2.09	0.105	0.76	0.50	1.15	0.190	0.89	0.65	1.21	0.440
TOP2A	1.21	1.11	1.32	**<0.001**	1.43	1.27	1.61	**<0.001**	1.21	0.99	1.49	0.067	0.92	0.70	1.22	0.584	0.91	0.70	1.17	0.460
UBE2C	1.25	1.11	1.40	**<0.001**	1.50	1.28	1.77	**<0.001**	1.49	1.07	2.08	**0.018**	0.64	0.42	0.95	**0.029**	1.15	0.87	1.51	0.323
**i-genes**																				
BTN3A2	0.93	0.80	1.08	0.345	0.91	0.73	1.13	0.409	1.08	0.71	1.63	0.723	0.67	0.44	1.01	0.057	1.05	0.77	1.45	0.740
CCL19	0.97	0.88	1.07	0.530	0.96	0.84	1.10	0.578	0.87	0.68	1.12	0.275	0.95	0.74	1.23	0.713	1.11	0.89	1.38	0.369
CD2	0.96	0.87	1.07	0.486	1.04	0.90	1.20	0.622	0.93	0.70	1.23	0.590	0.73	0.54	0.99	**0.040**	0.99	0.77	1.27	0.935
CD52	0.99	0.98	1.01	0.500	1.01	0.98	1.03	0.652	0.99	0.94	1.04	0.686	0.97	0.93	1.02	0.280	0.99	0.95	1.03	0.593
HLADPA1	0.98	0.88	1.09	0.762	0.96	0.82	1.11	0.545	0.85	0.65	1.11	0.239	0.91	0.70	1.17	0.462	1.22	0.94	1.59	0.142
TRBC1	0.93	0.80	1.08	0.344	0.93	0.74	1.16	0.521	0.92	0.61	1.39	0.690	0.74	0.52	1.06	0.100	1.10	0.80	1.52	0.559

Lymph node- negative

	All				HR+/HER2−				HR+/HER2+				HR−/HER2+				TNBC			
	Hazard ratio	95% CI		*P* value	Hazard ratio	95% CI		*P* value	Hazard ratio	95% CI		*P* value	Hazard ratio	95% CI		*P* value	Hazard ratio	95% CI		*P* value
**No. of patients (No. of events)**	422 (77)				203 (35)				45(8)				58(16)				116(18)			
**p-genes**																				
AURKA	1.06	0.94	1.20	0.363	1.22	1.02	1.47	**0.030**	0.95	0.62	1.46	0.809	0.96	0.72	1.30	0.813	0.97	0.76	1.23	0.794
CCNB2	1.17	0.93	1.48	0.188	1.44	1.00	2.06	**0.048**	1.10	0.57	2.12	0.787	0.79	0.41	1.53	0.486	1.20	0.64	2.24	0.566
FOXM1	1.27	1.04	1.54	**0.020**	1.57	1.17	2.11	**0.003**	2.29	0.95	5.48	0.064	0.89	0.43	1.85	0.752	1.11	0.66	1.85	0.702
MKI67	1.31	1.05	1.63	**0.018**	1.52	1.12	2.06	**0.008**	1.48	0.82	2.66	0.190	1.16	0.70	1.93	0.569	0.99	0.56	1.76	0.985
MMP11	1.31	1.12	1.54	**0.001**	1.35	1.08	1.70	**0.010**	1.86	1.10	3.14	**0.021**	1.40	0.95	2.06	0.091	1.02	0.72	1.43	0.917
PTTG1	1.15	0.85	1.54	0.364	1.19	0.76	1.85	0.451	1.51	0.74	3.05	0.256	0.82	0.41	1.66	0.589	1.27	0.65	2.48	0.483
RACGAP1	1.22	0.96	1.54	0.105	1.37	1.00	1.89	0.053	1.14	0.49	2.68	0.762	1.38	0.75	2.53	0.302	0.95	0.54	1.67	0.850
RRM2	1.32	1.04	1.67	**0.022**	1.79	1.26	2.55	**0.001**	1.26	0.50	3.19	0.627	0.80	0.43	1.47	0.467	1.03	0.62	1.72	0.915
TOP2A	1.25	1.07	1.46	**0.004**	1.48	1.20	1.84	**<0.001**	1.33	0.82	2.15	0.255	0.98	0.62	1.54	0.919	1.12	0.71	1.78	0.625
UBE2C	1.31	1.06	1.61	**0.012**	1.66	1.22	2.28	**0.001**	1.80	0.88	3.66	0.105	0.46	0.23	0.91	**0.026**	1.40	0.85	2.32	0.190

**i-genes**																				
BTN3A2	0.90	0.69	1.18	0.440	0.79	0.52	1.20	0.268	1.27	0.52	3.07	0.600	0.68	0.38	1.22	0.193	1.08	0.65	1.78	0.769
CCL19	0.94	0.80	1.11	0.490	0.85	0.66	1.08	0.174	0.64	0.36	1.12	0.118	0.98	0.66	1.45	0.913	1.29	0.91	1.82	0.146
CD2	0.91	0.76	1.08	0.276	0.82	0.63	1.07	0.142	0.82	0.44	1.53	0.541	0.80	0.53	1.21	0.300	1.09	0.74	1.59	0.659
CD52	0.99	0.95	1.02	0.476	0.99	0.94	1.05	0.840	0.99	0.89	1.11	0.884	0.95	0.86	1.03	0.223	1.00	0.95	1.06	0.988
HLADPA1	0.89	0.74	1.06	0.197	0.81	0.62	1.05	0.108	0.64	0.34	1.21	0.167	0.80	0.54	1.17	0.248	1.25	0.85	1.85	0.258
TRBC1	0.84	0.64	1.09	0.189	0.85	0.56	1.30	0.455	0.72	0.27	1.88	0.497	0.56	0.33	0.97	**0.038**	1.19	0.72	1.95	0.494

Lymph node-positive

	**All**				**HR+/HER2−**				**HR+/HER2+**				**HR−/HER2+**				**TNBC**			
	**Hazard ratio**	**95% CI**		***P* value**	**Hazard ratio**	**95% CI**		***P* value**	**Hazard ratio**	**95% CI**		***P* value**	**Hazard ratio**	**95% CI**		***P* value**	**Hazard ratio**	**95% CI**		***P* value**
**No. of patients (No. of events)**	395 (156)				207 (85)				66 (29)				46 (17)				76 (25)			
**p-genes**																				
AURKA	1.06	0.98	1.16	0.150	1.10	0.98	1.25	0.104	0.99	0.81	1.20	0.914	1.37	1.08	1.73	**0.009**	0.86	0.69	1.06	0.147
CCNB2	1.14	0.98	1.32	0.095	1.37	1.11	1.68	**0.003**	1.09	0.75	1.59	0.640	1.09	0.65	1.80	0.749	0.74	0.47	1.15	0.182
FOXM1	1.16	0.99	1.36	0.058	1.31	1.06	1.62	**0.014**	1.00	0.66	1.51	0.999	1.03	0.50	2.13	0.934	1.11	0.73	1.69	0.635
MKI67	1.26	1.06	1.50	**0.010**	1.53	1.16	2.01	**0.002**	1.27	0.85	1.92	0.245	1.17	0.72	1.91	0.525	0.96	0.60	1.56	0.880
MMP11	1.23	1.09	1.38	**0.001**	1.18	1.01	1.38	**0.035**	1.17	0.89	1.53	0.262	1.46	0.99	2.15	0.054	1.30	0.96	1.75	0.088
PTTG1	1.02	0.84	1.24	0.861	1.08	0.82	1.43	0.574	1.27	0.81	1.99	0.294	0.97	0.49	1.92	0.926	0.73	0.42	1.29	0.286
RACGAP1	1.08	0.91	1.29	0.359	1.15	0.92	1.44	0.229	1.11	0.69	1.79	0.661	0.87	0.48	1.59	0.659	1.03	0.68	1.56	0.878
RRM2	1.13	0.96	1.33	0.130	1.31	1.05	1.64	**0.015**	1.43	0.93	2.19	0.101	0.78	0.45	1.34	0.368	0.86	0.61	1.22	0.405
TOP2A	1.20	1.08	1.33	**0.001**	1.35	1.17	1.57	**<0.001**	1.24	0.99	1.56	0.061	0.89	0.63	1.26	0.507	0.92	0.68	1.24	0.592
UBE2C	1.21	1.06	1.39	**0.006**	1.38	1.14	1.66	**0.001**	1.38	0.94	2.01	0.101	0.82	0.48	1.39	0.460	1.04	0.77	1.39	0.813
**i-genes**																				
BTN3A2	1.00	0.83	1.20	0.999	1.05	0.81	1.35	0.728	1.09	0.68	1.75	0.729	0.68	0.36	1.26	0.220	1.06	0.71	1.56	0.786
CCL19	0.97	0.86	1.09	0.582	1.01	0.86	1.19	0.916	0.92	0.69	1.22	0.556	0.91	0.68	1.22	0.549	0.98	0.73	1.32	0.897
CD2	0.99	0.87	1.14	0.925	1.14	0.95	1.36	0.149	0.87	0.63	1.22	0.425	0.66	0.42	1.03	0.070	0.93	0.67	1.31	0.693
CD52	0.99	0.97	1.02	0.648	1.01	0.98	1.04	0.695	0.99	0.94	1.05	0.798	0.99	0.94	1.05	0.815	0.98	0.93	1.04	0.478
HLADPA1	1.03	0.90	1.17	0.664	1.01	0.84	1.20	0.956	0.94	0.69	1.29	0.715	0.98	0.69	1.39	0.897	1.27	0.86	1.86	0.233
TRBC1	0.94	0.78	1.13	0.520	0.93	0.72	1.21	0.592	1.02	0.65	1.59	0.935	0.86	0.51	1.47	0.591	1.01	0.66	1.55	0.961

Abbreviations: HR, hormone receptor; HER2, human epidermal growth factor receptor 2; TNBC, triple-negative breast cancer; CI, confidence interval; p-genes, proliferation-related genes; i-genes, immune response-related genes. Hazard ratios with *P* values < 0.05 are marked in bold.

Supplementary Table S3. Univariate analysis of gene variables for OS according to molecular subtype

	All				HR+/HER2−				HR+/HER2+				HR−/HER2+				TNBC			
	Hazard ratio	95% CI		*P* value	Hazard ratio	95% CI		*P* value	Hazar d ratio	95% CI		*P* value	Hazard ratio	95% CI		*P* value	Hazard ratio	95% CI		*P* value
**No. of patients (No. of events)**	815 (195)				410 (94)				110 (31)				105 (27)				190 (43)			
**p-genes**																				
AURKA	1.05	0.97	1.14	0.192	1.15	1.02	1.28	**0.018**	0.86	0.69	1.06	0.145	1.14	0.93	1.39	0.215	0.94	0.80	1.10	0.429
CCNB2	1.19	1.03	1.37	**0.015**	1.36	1.11	1.67	**0.003**	1.25	0.89	1.77	0.201	1.23	0.78	1.94	0.376	0.81	0.57	1.16	0.258
FOXM1	1.21	1.06	1.38	**0.004**	1.36	1.13	1.63	**0.001**	1.23	0.81	1.85	0.329	1.31	0.78	2.19	0.310	0.99	0.71	1.36	0.928
MKI67	1.19	1.03	1.38	**0.021**	1.39	1.13	1.71	**0.002**	1.21	0.86	1.71	0.281	1.14	0.77	1.69	0.512	0.79	0.54	1.17	0.248
MMP11	1.17	1.06	1.30	**0.002**	1.09	0.95	1.26	0.218	1.17	0.92	1.49	0.208	1.48	1.10	2.00	**0.009**	1.17	0.94	1.46	0.166
PTTG1	1.02	0.85	1.21	0.859	1.04	0.79	1.35	0.796	1.25	0.83	1.87	0.290	1.09	0.65	1.84	0.746	0.75	0.48	1.18	0.217
RACGAP1	1.13	0.97	1.31	0.127	1.23	1.00	1.51	0.053	1.37	0.89	2.11	0.156	1.07	0.68	1.67	0.781	0.83	0.59	1.18	0.309
RRM2	1.20	1.04	1.39	**0.013**	1.41	1.14	1.74	**0.002**	1.69	1.06	2.68	**0.027**	0.89	0.57	1.38	0.604	0.92	0.67	1.25	0.584
TOP2A	1.19	1.08	1.31	**<0.001**	1.40	1.22	1.60	**<0.001**	1.07	0.86	1.33	0.547	1.04	0.77	1.39	0.812	0.88	0.69	1.13	0.331
UBE2C	1.29	1.13	1.46	**<0.001**	1.58	1.32	1.90	**<0.001**	1.31	0.90	1.89	0.159	0.87	0.56	1.33	0.515	1.04	0.80	1.36	0.757
**i-genes**																				
BTN3A2	0.90	0.76	1.06	0.193	0.94	0.74	1.20	0.637	1.13	0.72	1.78	0.589	0.52	0.33	0.84	**0.007**	0.96	0.71	1.31	0.797
CCL19	0.96	0.87	1.07	0.479	0.97	0.83	1.13	0.680	0.97	0.74	1.28	0.845	0.88	0.68	1.14	0.337	1.01	0.81	1.26	0.920
CD2	0.96	0.86	1.07	0.447	1.12	0.96	1.32	0.155	0.84	0.63	1.13	0.249	0.65	0.46	0.90	**0.011**	0.88	0.69	1.12	0.284
CD52	0.99	0.97	1.01	0.311	1.01	0.98	1.04	0.687	0.99	0.93	1.05	0.673	0.97	0.91	1.02	0.215	0.98	0.94	1.02	0.267
HLADPA1	1.03	0.91	1.15	0.665	0.98	0.83	1.17	0.851	1.08	0.79	1.48	0.617	0.90	0.68	1.20	0.473	1.16	0.89	1.52	0.257
TRBC1	0.92	0.78	1.08	0.305	1.06	0.83	1.35	0.654	0.92	0.60	1.40	0.687	0.63	0.42	0.94	**0.023**	0.96	0.69	1.32	0.788

Lymph node- negative

	All				HR+/HER2−				HR+/HER2+				HR−/HER2+				TNBC			
	Hazard ratio	95% CI		*P* value	Hazard ratio	95% CI		*P* value	Hazard ratio	95% CI		*P* value	Hazard ratio	95% CI		*P* value	Hazard ratio	95% CI		*P* value
**No. of patients (No. of events)**	422 (61)				203 (24)				45 (9)				59 (13)				115 (15)			
**p-genes**																				
AURKA	1.06	0.92	1.21	0.452	1.10	0.86	1.39	0.460	0.89	0.59	1.34	0.578	1.01	0.72	1.40	0.963	1.04	0.80	1.35	0.753
CCNB2	1.20	0.92	1.56	0.175	1.56	1.02	2.39	**0.041**	0.99	0.55	1.79	0.975	0.86	0.41	1.80	0.690	1.03	0.53	2.01	0.931
FOXM1	1.38	1.11	1.73	**0.005**	1.78	1.26	2.50	**0.001**	1.47	0.67	3.23	0.339	1.08	0.51	2.31	0.840	1.20	0.68	2.12	0.520
MKI67	1.12	0.86	1.46	0.386	1.61	1.14	2.28	**0.007**	1.04	0.56	1.94	0.900	0.82	0.45	1.48	0.500	0.62	0.31	1.26	0.190
MMP11	1.20	1.01	1.42	**0.040**	1.12	0.86	1.47	0.408	1.31	0.84	2.05	0.240	1.37	0.91	2.05	0.130	1.09	0.75	1.58	0.645
PTTG1	0.99	0.71	1.39	0.960	1.04	0.61	1.78	0.872	1.01	0.47	2.18	0.974	0.82	0.38	1.78	0.615	1.11	0.53	2.32	0.781
RACGAP1	1.04	0.79	1.36	0.801	1.24	0.84	1.85	0.284	0.94	0.39	2.28	0.892	1.03	0.56	1.90	0.922	0.73	0.38	1.38	0.329
RRM2	1.39	1.06	1.81	**0.015**	1.95	1.27	3.00	**0.002**	0.84	0.37	1.93	0.687	1.01	0.52	1.97	0.977	1.16	0.67	2.03	0.594
TOP2A	1.27	1.07	1.51	**0.007**	1.49	1.15	1.94	**0.003**	1.07	0.71	1.61	0.749	1.18	0.77	1.82	0.452	1.01	0.62	1.65	0.979
UBE2C	1.43	1.13	1.82	**0.003**	2.17	1.47	3.20	**<0.001**	1.42	0.70	2.87	0.334	0.59	0.30	1.19	0.141	1.29	0.75	2.22	0.366

**i-genes**																				
BTN3A2	0.82	0.61	1.11	0.202	0.92	0.55	1.53	0.747	1.01	0.46	2.23	0.987	0.43	0.22	0.84	**0.013**	1.03	0.59	1.78	0.929
CCL19	0.90	0.75	1.09	0.271	0.84	0.62	1.12	0.234	0.82	0.49	1.40	0.474	0.83	0.53	1.28	0.394	1.13	0.77	1.65	0.534
CD2	0.88	0.73	1.07	0.207	0.89	0.65	1.23	0.496	0.76	0.42	1.38	0.367	0.74	0.47	1.16	0.187	0.89	0.59	1.35	0.590
CD52	0.99	0.95	1.03	0.463	1.00	0.94	1.07	0.993	1.00	0.90	1.12	0.970	0.94	0.85	1.04	0.254	0.98	0.92	1.05	0.607
HLADPA1	0.94	0.76	1.15	0.532	0.88	0.63	1.23	0.453	0.86	0.48	1.53	0.607	0.83	0.54	1.27	0.389	1.08	0.70	1.66	0.717
TRBC1	0.74	0.54	1.00	0.051	1.10	0.67	1.80	0.703	0.50	0.19	1.33	0.165	0.37	0.19	0.71	**0.003**	0.97	0.56	1.68	0.914

Lymph node- positive

	**All**				**HR+/HER2−**				**HR+/HER2+**				**HR−/HER2+**				**TNBC**			
	**Hazard ratio**	**95% CI**		***P* value**	**Hazard ratio**	**95% CI**		***P* value**	**Hazard ratio**	**95% CI**		***P* value**	**Hazard ratio**	**95% CI**		***P* value**	**Hazard ratio**	**95% CI**		***P* value**
**No. of patients (No. of events)**	393 (134)				207 (70)				65 (22)				46 (14)				75 (28)			
**p-genes**																				
AURKA	1.03	0.94	1.13	0.471	1.11	0.98	1.27	0.101	0.81	0.64	1.04	0.097	1.23	0.95	1.58	0.112	0.89	0.73	1.08	0.252
CCNB2	1.21	1.03	1.42	**0.021**	1.30	1.03	1.63	**0.026**	1.42	0.91	2.23	0.124	1.46	0.82	2.60	0.196	0.74	0.50	1.10	0.139
FOXM1	1.20	1.02	1.41	**0.032**	1.18	0.95	1.46	0.139	1.15	0.70	1.88	0.582	1.83	0.80	4.18	0.153	1.07	0.72	1.60	0.735
MKI67	1.35	1.12	1.62	**0.002**	1.48	1.12	1.97	**0.006**	1.55	0.93	2.58	0.093	1.54	0.97	2.45	0.068	0.88	0.54	1.41	0.585
MMP11	1.12	0.99	1.27	0.072	1.03	0.87	1.22	0.718	1.11	0.83	1.49	0.472	1.58	1.02	2.45	**0.039**	1.21	0.92	1.60	0.177
PTTG1	1.10	0.90	1.36	0.349	1.16	0.85	1.59	0.339	1.47	0.88	2.44	0.142	1.41	0.70	2.84	0.337	0.66	0.38	1.15	0.142
RACGAP1	1.20	0.99	1.45	0.063	1.20	0.93	1.54	0.165	1.64	0.94	2.86	0.084	1.10	0.56	2.18	0.778	1.00	0.68	1.47	0.992
RRM2	1.15	0.97	1.37	0.103	1.23	0.97	1.55	0.088	2.03	1.23	3.34	**0.006**	0.86	0.48	1.55	0.618	0.89	0.63	1.26	0.504
TOP2A	1.16	1.04	1.30	**0.007**	1.30	1.11	1.52	**0.001**	1.09	0.84	1.42	0.523	0.95	0.65	1.38	0.794	0.98	0.74	1.30	0.902
UBE2C	1.23	1.06	1.42	**0.007**	1.38	1.12	1.70	**0.003**	1.23	0.79	1.93	0.357	1.22	0.68	2.17	0.505	0.99	0.76	1.30	0.942
**i-genes**																				
BTN3A2	0.98	0.81	1.19	0.871	1.04	0.79	1.37	0.780	1.22	0.69	2.13	0.492	0.63	0.31	1.29	0.204	0.96	0.68	1.36	0.805
CCL19	0.98	0.86	1.11	0.766	1.01	0.85	1.20	0.909	1.00	0.72	1.39	0.995	0.90	0.65	1.23	0.504	0.93	0.71	1.23	0.622
CD2	1.00	0.86	1.15	0.957	1.22	1.00	1.48	0.050	0.83	0.59	1.17	0.291	0.57	0.35	0.92	**0.023**	0.87	0.64	1.18	0.378
CD52	0.99	0.96	1.01	0.379	1.00	0.97	1.04	0.810	0.98	0.92	1.06	0.666	0.98	0.92	1.05	0.513	0.97	0.92	1.02	0.273
HLADPA1	1.06	0.92	1.23	0.393	1.00	0.83	1.22	0.972	1.18	0.79	1.76	0.407	0.94	0.64	1.39	0.773	1.29	0.89	1.87	0.177
TRBC1	0.98	0.80	1.19	0.822	1.01	0.76	1.35	0.943	1.12	0.68	1.82	0.659	0.93	0.52	1.66	0.803	0.89	0.59	1.34	0.581

Abbreviations: HR, hormone receptor; HER2, human epidermal growth factor receptor 2; TNBC, triple-negative breast cancer; CI, confidence interval; p-genes, proliferation-related genes; i-genes, immune response-related genes. Hazard ratios with *P* values < 0.05 are marked in bold.

Supplementary Table S4. Multivariate analysis of DFS and OS according to molecular subtype

	All				HR+/HER2−				HR+/HER2+				HR−/HER2+				TNBC			
	**Hazard ratio**	**95% CI**		***P* value**	**Hazard ratio**	**95% CI**		***P* value**	**Hazard ratio**	**95% CI**		***P* value**	**Hazard ratio**	**95% CI**		***P* value**	**Hazard ratio**	**95% CI**		***P* value**
**DFS**																				
**No. of patients (No. of events)**	817 (233)				410 (120)				111 (37)				104 (33)				192 (43)			
**Age**																				
<50	1.00								1.00											
≥50	0.81	0.62	1.07	0.137					0.33	0.13	0.80	**0.014**								
**Lymph node status**																				
Negative	1.00				1.00				1.00								1.00			
Positive	2.38	1.80	3.14	**<0.001**	2.47	1.65	3.70	**<0.001**	2.98	1.36	6.55	**0.006**					2.50	1.37	4.59	**0.003**
**p-genes**																				
MKI67	1.12	0.97	1.31	0.129	1.22	0.98	1.52	0.079												
MMP11	1.24	1.13	1.36	**<0.001**	1.17	1.03	1.34	**0.020**	1.25	0.98	1.61	0.073	1.46	1.11	1.92	**0.006**				
TOP2A	1.17	1.07	1.29	**0.001**	1.32	1.15	1.50	**<0.001**												
UBE2C									1.52	1.05	2.20	**0.026**	0.62	0.41	0.94	**0.024**				
**OS**																				
**No. of patients (No. of events)**	815 (195)				410 (94)				110 (31)				105 (27)				190 (43)			
**Tumor size (cm)**																				
≤2	1.00																			
2-5	1.18	0.83	1.66	0.353																
>5	2.07	1.30	3.30	**0.002**																
**Lymph node status**																				
Negative	1.00				1.00												1.00			
Positive	2.25	1.63	3.11	**<0.001**	2.91	1.81	4.68	**<0.001**									3.44	1.84	6.45	**<0.001**
**Histologic grade**																				
1	1.00																			
2	1.44	0.73	2.83	0.291																
3	1.86	0.95	3.64	0.071																
**p-genes**																				
MKI67					1.22	0.94	1.58	0.127												
MMP11	1.11	1.00	1.24	0.052									1.47	1.05	2.06	**0.025**				
RRM2									1.69	1.06	2.68	**0.027**								
TOP2A					1.17	0.99	1.39	0.066												
UBE2C	1.23	1.07	1.41	**0.003**	1.23	0.97	1.57	0.088												
**i-genes**																				
BTN3A2	0.85	0.67	1.07	0.159	0.77	0.55	1.07	0.116					0.56	0.34	0.92	**0.023**				

Abbreviations: HR, hormone receptor; HER2, human epidermal growth factor receptor 2; TNBC, triple-negative breast cancer; CI, confidence interval; p-genes, proliferation-related genes; i-genes, immune response-related genes. Hazard ratios with *P* values < 0.05 are marked in bold.

Supplementary Table S5. Clinical characteristics of low-risk and high-risk defined by our risk score to predict the risk of distant metastasis in HR−/HER2+ breast cancer

	Low-risk group	High-risk group	*P* value
**No. of samples**	52	52	
**Age (median, years)**	51.6	51.3	0.883[a]
**Tumor size (mean, cm)**	3.01	3.05	0.909[a]
**Tumor size (cm)**			0.409[b]
≤2	15	17	
2-5	30	32	
>5	7	3	
**Lymph node status**			0.844[b]
Negative	30	28	
Positive	22	24	
**Pathologic stage**			0.973[b]
I	10	10	
II	29	28	
III	13	14	
**Histologic grade**			0.290[c]
1	1	3	
2	10	13	
3	38	28	
NA	3	8	
**Chemotherapy**			1.000[b]
No	7	6	
Yes	45	45	
NA	0	1	
**NPI**			0.342[c]
1	2	5	
2	13	14	
3	27	17	
4	7	8	
NA	3	8	

a: Student's t-test; b: Chi-square test; c: Fisher's exact test
Abbreviations: NPI, Nottingham prognostic index, *P* values < 0.05 are marked in bold.

Supplementary Figures

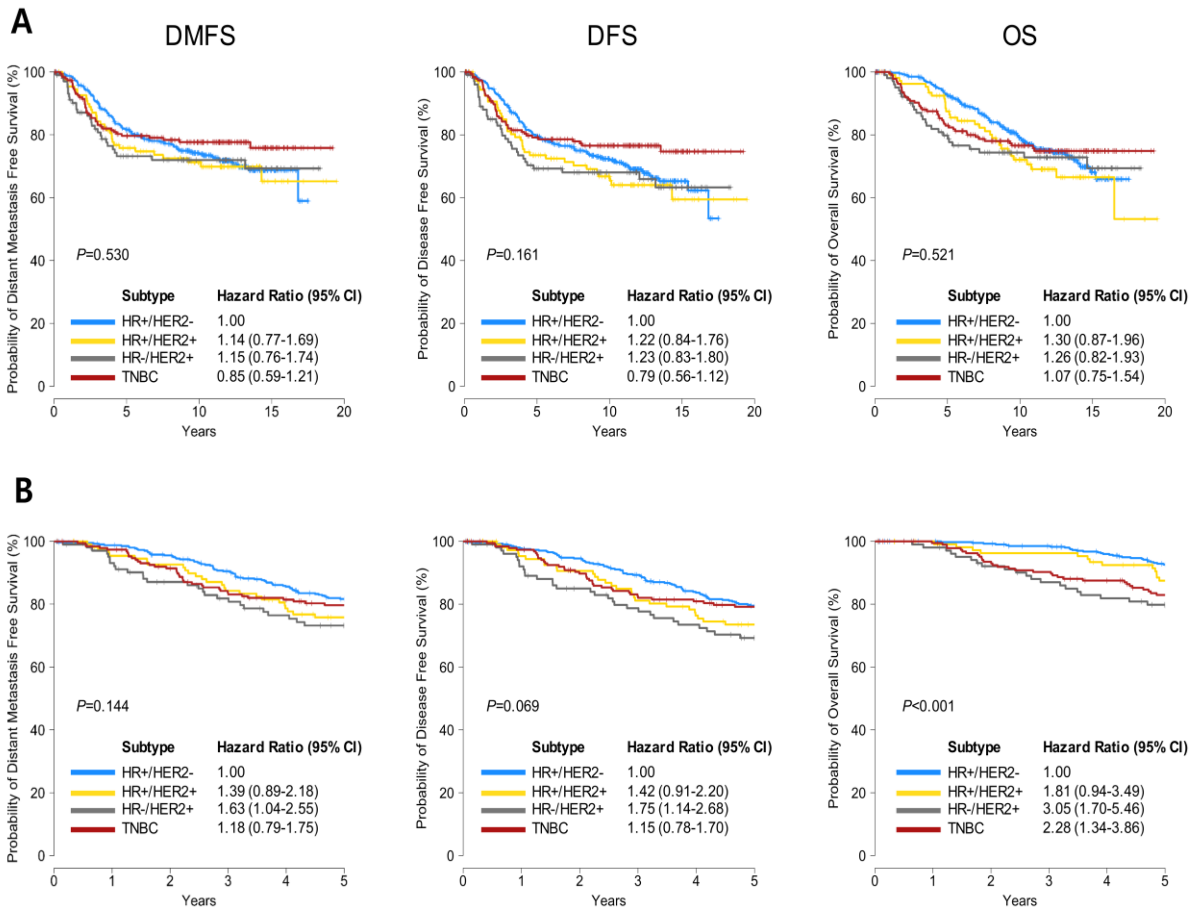

Supplementary Fig. S1. Patient outcomes according to molecular subtypes. Kaplan-Meier plots comparing the probability of distant metastasis-free survival (DMFS), disease-free survival (DFS), and overall survival (OS) of patients classified as four molecular subtypes (A) during entire follow-up period and (B) 5-year follow-up period. *P* value < 0.05 indicates that there are differences in survival between the subtypes. The hazard ratios describe the relative risk associated with each molecular subtype compared with the HR+/HER2- subtype. CI, confidence interval; HR, hormone receptor; HER2, human epidermal growth factor receptor 2; TNBC, triple-negative breast cancer.

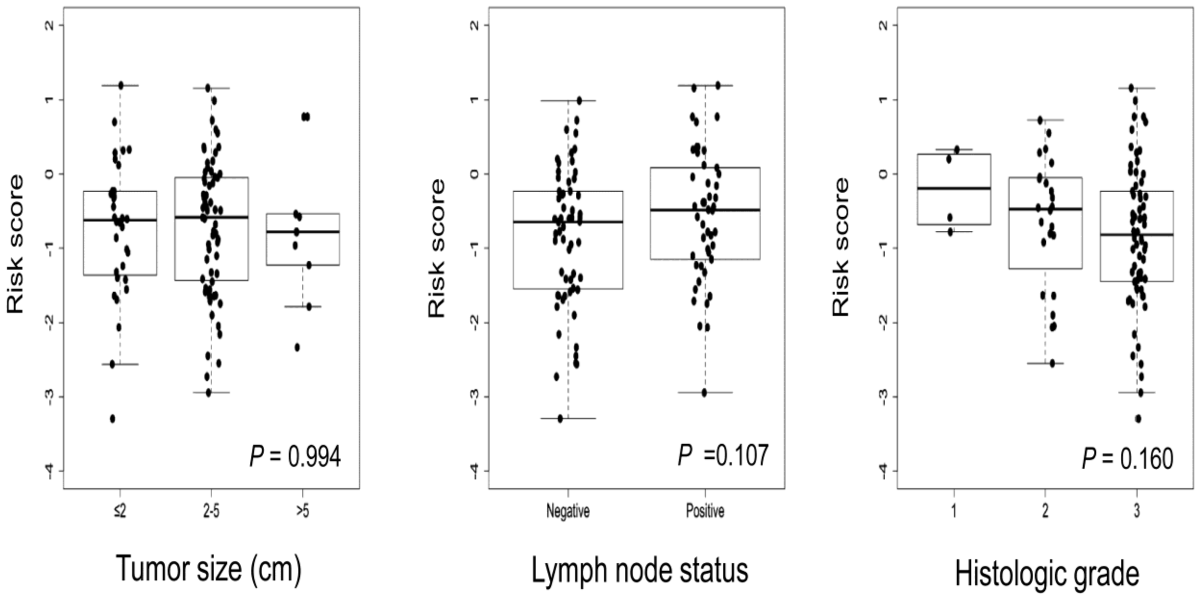

Supplementary Fig. S2. Association between our risk score and traditional clinicopathological parameters in HR−/HER2+ breast cancer. Statistical significances were calculated by one way ANOVA or Student's t-test.

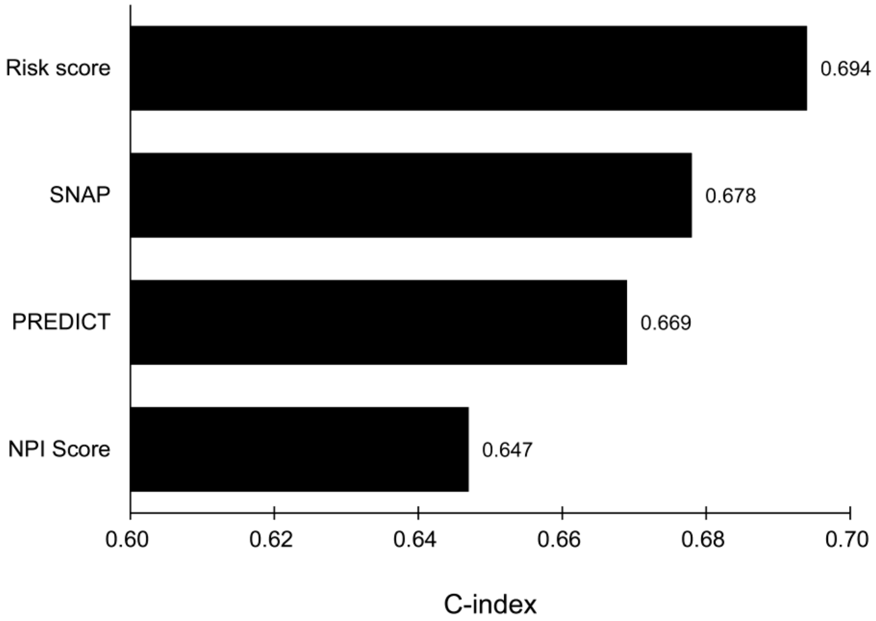

Supplementary Fig. S3. C-index indicating the prognostic performance of our risk score and other prognostic models based on clinicopathological parameters alone. Values on the x-axis are unbiased estimates of the C-index of the linear combination of one or more variables by Cox regression. PREDICT (www.predict.nhs.uk); SNAP (www.CancerMath.net); NPI, Nottingham prognostic index.
